# Tephritid Fruit Fly Semiochemicals: Current Knowledge and Future Perspectives

**DOI:** 10.3390/insects12050408

**Published:** 2021-04-30

**Authors:** Francesca Scolari, Federica Valerio, Giovanni Benelli, Nikos T. Papadopoulos, Lucie Vaníčková

**Affiliations:** 1 Institute of Molecular Genetics IGM-CNR “Luigi Luca Cavalli-Sforza”, I-27100 Pavia, Italy; 2 Department of Biology and Biotechnology, University of Pavia, I-27100 Pavia, Italy; federica.valerio02@universitadipavia.it; 3 Department of Agriculture, Food and Environment, University of Pisa, Via del Borghetto 80, 56124 Pisa, Italy; giovanni.benelli@unipi.it; 4 Department of Agriculture Crop Production and Rural Environment, University of Thessaly, Fytokou st., N. Ionia, 38446 Volos, Greece; nikopap@uth.gr; 5 Department of Chemistry and Biochemistry, Faculty of AgriSciences Mendel University in Brno, Zemedelska 1, CZ-613 00 Brno, Czech Republic; 6 Department of Forest Botany, Dendrology and Geobiocoenology, Faculty of Forestry and Wood Technology, Mendel University in Brno, Zemedelska 1, CZ-613 00 Brno, Czech Republic

**Keywords:** pheromone, olfactory cues, mating disruption, cuticular hydrocarbons, host-marking pheromone, true fruit flies, olfaction, odours

## Abstract

**Simple Summary:**

Tephritid fruit flies comprise pests of high agricultural relevance and species that have emerged as global invaders. Chemical signals play key roles in multiple steps of a fruit fly’s life. The production and detection of chemical cues are critical in many behavioural interactions of tephritids, such as finding mating partners and hosts for oviposition. The characterisation of the molecules involved in these behaviours sheds light on understanding the biology and ecology of fruit flies and in addition provides a solid base for developing novel species-specific pest control tools by exploiting and/or interfering with chemical perception. Here we provide a comprehensive overview of the extensive literature on different types of chemical cues emitted by tephritids, with a focus on the most relevant fruit fly pest species. We describe the chemical identity, production modality and behavioural relevance of volatile pheromones, host-marking pheromones and cuticular hydrocarbons, as well as the technological advances available for their characterisation. The variegate set of approaches integrating the use of the identified chemical signals for the control of wild populations of key pests is also explored. Last but not least, key challenges for future basic to applied research regarding tephritids are outlined.

**Abstract:**

The Dipteran family Tephritidae (true fruit flies) comprises more than 5000 species classified in 500 genera distributed worldwide. Tephritidae include devastating agricultural pests and highly invasive species whose spread is currently facilitated by globalization, international trade and human mobility. The ability to identify and exploit a wide range of host plants for oviposition, as well as effective and diversified reproductive strategies, are among the key features supporting tephritid biological success. Intraspecific communication involves the exchange of a complex set of sensory cues that are species- and sex-specific. Chemical signals, which are standing out in tephritid communication, comprise long-distance pheromones emitted by one or both sexes, cuticular hydrocarbons with limited volatility deposited on the surrounding substrate or on the insect body regulating medium- to short-distance communication, and host-marking compounds deposited on the fruit after oviposition. In this review, the current knowledge on tephritid chemical communication was analysed with a special emphasis on fruit fly pest species belonging to the *Anastrepha, Bactrocera,* *Ceratitis*, *Rhagoletis* and *Zeugodacus* genera. The multidisciplinary approaches adopted for characterising tephritid semiochemicals, and the real-world applications and challenges for Integrated Pest Management (IPM) and biological control strategies are critically discussed. Future perspectives for targeted research on fruit fly chemical communication are highlighted.

## 1. Introduction

Insect semiochemicals are compounds belonging to different chemical classes that regulate intra- and inter-specific communication, affecting major behavioural and physiological responses [1,2,3]. Based on the identity of the emitter and the receiver, semiochemicals can be classified as pheromones (i.e., molecules mediating communication between co-specifics) or allelochemicals (i.e., compounds involved in communication between individuals of different species). Allelochemicals include kairomones (beneficial to the receiver but producing disadvantages for the emitter), synomones (molecules that benefit both the emitter and the receiver), allomones (beneficial to the producer and with neutral effects to the receiver), and apneumones (chemicals of non-biological origin beneficial to the receiver) [4,5,6]. Semiochemicals mediate a number of behavioural processes, such as the identification of food sources, the location of mates and hosts for oviposition, and the avoidance of predators [7].

To achieve these different functions, insects use volatile, semi-volatile, and non-volatile chemicals that are involved in long-, medium-, and short-distance communication, respectively. These stimuli can be detected by sensory neurons of the olfactory system on the antennae and maxillary palps [8], such as in the case of volatile molecules, or by neurons of the gustatory system mainly on proboscis, ovipositor and legs, which are able to perceive non-volatile chemicals through contact chemoreception [9].

Among semiochemicals, pheromones (from the Greek words “φερειν”-transfer, and “ορμαν”-excite) can be classified in two main categories: (*i*) releaser pheromones that produce an immediate response in a recipient individual (e.g., a male fly orienting toward a female guided by sex pheromone), (*ii*) primer pheromones that trigger the initiation of a complex physiological response not immediately observable [10]. The complex functions pheromones exert are mediated by sex, aggregation, alarm, trails and host-marking compounds [11,12].

Since 1959, when the term ‘pheromone’ was proposed [13] and the first pheromone, bombykol, was chemically identified in the silkworm moth, *Bombyx mori* (L.) (Lepidoptera: Bombycidae) [14], an increasing number of studies have focused on unravelling the chemistry and biological roles of these substances in numerous species. In most cases, pheromones are blends of individual chemicals that can be shared among species, but that are mixed in species-specific combinations (i.e., quantitatively and qualitatively) [15,16]. So far, volatile pheromones have been described as being composed of two or three compounds in moths [17,18], one in *Drosophila melanogaster* Meigen (Diptera: Drosophilidae) ((*Z*)-undec-4-enal [19]), or by complex blends in honey bees (Hymenoptera: Apidae) [20]. The chemical diversity of pheromone blends is very high, including acetate esters, alcohols, aldehydes, carboxylic acids, hydrocarbons, epoxides, ketones, benzenoid compounds, isoprenoids, terpenoids, and triacylglycerides [21]. In the case of Lepidoptera, as well as other insects that mainly rely on long-distance sexual signalling, volatile pheromones are the primary semiochemicals adopted [22]. Other insects, such as *Drosophila* species, are characterised by complex courtship rituals and use cuticular hydrocarbons (CHs) of both high and low-volatility [23]. The body surface of many insect species is indeed covered by a thin film of wax, composed mainly of hydrocarbons. Complex mixtures of esters, alcohols and free fatty acids are components of the cuticular wax in some insects [24]. Beside their cuticle waterproofing function, the long-chain hydrocarbons of insects are involved in chemical communication, serving as sex pheromones, kairomones, species- and gender-recognition cues, nestmate recognition compounds, fertility and dominance cues, chemical mimicry, and primer pheromones. Such key roles boosted research efforts in the past several decades on many dipteran species, including fruit flies, house flies and mosquitoes [25,26]. Insect CHs are usually a mixture of compounds that may include *n*-alkanes, alkenes, terminally branched monomethylalkanes, internally branched monomethylalkanes, dimethylalkanes, trimethylalkanes and others. They are synthesised by an elongation-decarboxylation pathway in oenocytes, which are associated with epidermal cells or fat bodies. After synthesis, CHs are transported through haemolymph by lipophorin carrier [27,28].

An additional type of semiochemicals is used by several parasitic and phytophagous insects immediately after egg-laying, the host-marking pheromone (HMP). Its function is to affect the oviposition behaviour of conspecifics in a way that subsequent eggs are not deposited in their already utilised resource, thus reducing the time spent on the already exploited resource and the competition for limited host resources, with advantages for both the marker and the seeker [29]. The HMP can be synthetized by female fruit flies in the form of a complex molecule [30,31,32] or a simple compound [33,34,35]. The receptors located in the tarsi and mouthparts of females searching for an oviposition site allow the detection of HMPs [29,36,37].

In the last decades, progress has been made to determine the identity and composition of semiochemicals in insects, as well as the chemical specificity and functional properties of molecules mediating semiochemical perception such as odour and taste receptors, odorant and gustatory binding proteins. Insects developed extremely refined abilities to produce and discriminate among different arrays of chemicals. In this framework, an increasingly deeper knowledge of the mechanisms underlying semiochemicals’ production and stimuli coding is being acquired, also due to advancements in analytical approaches, which, in turn, is providing multiple novel/improved tools for insect pest control.

**Figure 1:**
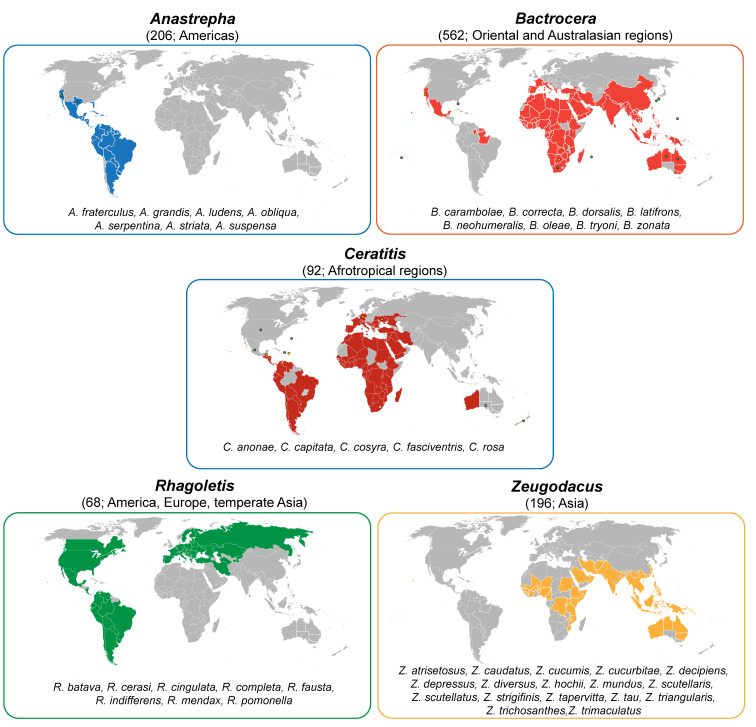
Worldwide distribution of species belonging to the *Anastrepha*, *Bactrocera*, *Ceratitis*, *Rhagoletis* and *Zeugodacus* genera. The map is based on the currently available distribution data of species belonging to each genus retrieved from the EPPO Global Database (https://gd.eppo.int) (accessed on 13 November 2020) and integrated with information from CABI (https://www.cabi.org/isc/) (accessed on 13 November 2020) and the literature. In brackets, the total number of living species (obtained from Catalogue of Life: 2019 Annual Checklist [40] and [39]) for each genus and the original geographic range are reported. Lists of the major pests within each genus are also indicated. Background map was retrieved from https://freevectormaps.com/world-maps/WRLD-EPS-01-0011?ref=atr (accessed on 29 July 2020). Information about the status of invasion is reported in more details for the species *A. ludens*, *B. dorsalis* and *C. capitata*, for which yellow and green dots indicate transient presence or achieved eradication, respectively.

In the scenario depicted above, true fruit flies (Diptera: Tephritidae) are excellent models to investigate the differentiation of semiochemicals’ production and perception. Including more than 5000 species and 500 genera, this family is one of the largest among dipteran, with a worldwide distribution. Many tephritids are important pests of agricultural commodities infesting a wide range of fruits and vegetables. The most pestiferous species belong to genera *Anastrepha* Schiner, *Bactrocera* Macquart, *Ceratitis* Macleay, *Rhagoletis* Loew, and *Zeugodacus* Hendel [38,39] (Figure 1).

As an example, the damage caused annually in Africa by the Oriental fruit fly, *Bactrocera dorsalis* (Hendel), has been estimated to USD 2 billion mainly due to export trade bans [41]. Moreover, due to a number of biological features including multivoltinism, long adult longevity, high fecundity, remarkable response to various stresses, increased capacity to overwinter [42], several species became aggressive global invaders, imposing strict quarantine regulations in several fruit-producing countries [43,44]. Because of their high importance, there is a list of ongoing programs worldwide that aim to eradicate, contain or suppress the populations of tephritid species [45].

Area-Wide Integrated Pest Management (AW-IPM) has been proven successful for the control of tephritid pest species and incorporates different components, such as thorough population monitoring employing sophisticated trapping systems, the Sterile Insect Technique (SIT), the Male Annihilation Technique (MAT) and often bait insecticidal sprayings [46]. To ensure effective application of the above components of the AW-IPM programs, an in-depth understanding of insect communication and mating strategies is required. This field has been widely investigated in tephritids and an expanding body of literature is available.

In this review, we critically discuss the current knowledge on pheromone-based communication in tephritid fruit flies, as well as its applied relevance for pest control. Starting from the role of these semiochemicals in tephritid reproductive behaviour, we analysed the most relevant, available literature focusing on (*i*) volatile pheromones released by males and/or females, (*ii*) HMPs, and (*iii*) CHs. For each of these three groups, the tissues involved in the production, their chemical identity, and the analytical methods applied for the identification, as well as the electrophysiological and behavioural tools employed to shed light on their ecological significance, are considered. Due to their high economic importance, insights are provided on pest species of the genera *Anastrepha, Bactrocera, Ceratitis*, *Rhagoletis* and *Zeugodacus*. Over the last decades, tephritid semiochemical research has not been equally dedicated to all three groups of semiochemicals. Thus, information related to volatile pheromones, mostly because of their more direct applied implications, is far more extended. This is also reflected in the present review, which additionally focuses on the role of semiochemical-based communication in species evolution. Tephritids display different levels of host specialisation, covering the whole spectrum from monophagy to polyphagy. Whether and how the adaptation to novel host plants drove the evolution of such an extreme diversity is still an open question and requires investigation of multiple factors (and their interactions), including those related to semiochemical-based communication. Finally, results focusing on applying the above-mentioned knowledge to fruit fly population monitoring and management, as well as to chemical taxonomy, are analysed, towards formulating major challenges for future research.

## 2. Semiochemicals and Reproductive Behaviour—An Overview

**Figure 2:**
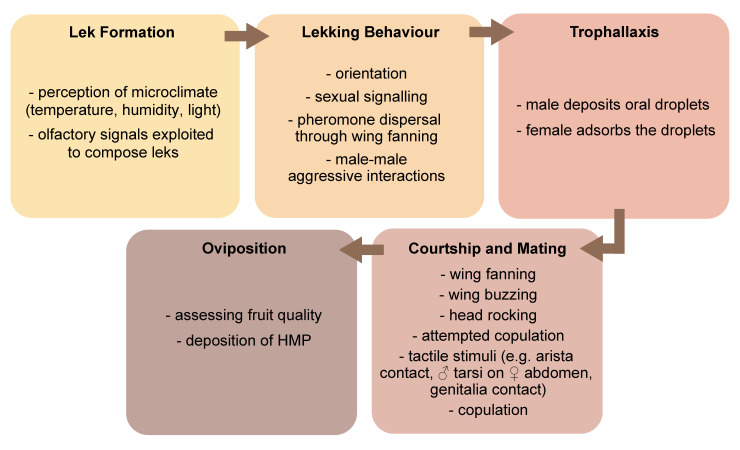
Diagram showing the main steps of mating behaviour in tephritids and the signals transmitted and perceived. Lekking behaviour is common in Tephritidae, but notably absent in *Rhagoletis* species. Similarly, pre-mating trophallaxis is common, but this phenomenon is known to occur also during and post mating [47]. HMP, host-marking pheromone.

Semiochemicals are involved in the different phases of the reproductive behaviour of many tephritids, which include male lek formation and sexual signalling (sexual calling), courtship, acceptance of a mating partner and successful copulation (Figure 2).

The release and perception of semiochemicals are often accompanied by a range of intense behavioural interactions, including wing vibration and buzzing and head rocking [48,49,50,51].

There is a variety of mating systems in tephritid fruit flies, with lek-based ones being common in many species of economic importance [52]. Leks are mating arenas where males aggregate and perform sexual signalling without controlling the access to resources that may be critical for females or offering parental care, and provide only the sperm to females that freely choose their mates [53]. Males perform sexual signalling mainly on the under surface of leaves of preferred host trees that either bear fruits as in *Anastrepha fraterculus* (Wiedemann) and *B. dorsalis*, or do not bear fruits as in certain populations of the Mediterranean fruit fly (medfly) *Ceratitis capitata* (Wiedemann) (see [54] for a review). Instead, in *Zeugodacus cucurbitae* (Coquillett), lekking takes place more frequently on non-host plants [55,56]. Likewise, in *Bactrocera cacuminata* (Hering), the wild tobacco fruit fly, non-host plants containing the male attractant methyl eugenol (ME) serve as the main mating sites [57]. Lekking males, in groups of 2–10 individuals [54], perform sexual signalling that includes a set of visual, acoustic and olfactory signals, with volatile pheromone acting as the longer-distance cue.

On the other hand, at variance with many tephritids, most *Rhagoletis* spp. do not exhibit male lekking [52]. For example, males of *Rhagoletis pomonella* (Walsh), *Rhagoletis cerasi* (L.), *Rhagoletis ribicola* Doane and *Rhagoletis mendax* Curran individually search for potential mates mainly on host fruits and the top surface of leaves [58], mostly during the late afternoon and at dusk [52,59,60]. However, in *Rhagoletis batava* Hering individuals have been reported to form small groups in response to male-released pheromone that might be considered leks [61]. While olfactory cues seem not to play a role in lek location for *Anastrepha obliqua* (Macquart) and *Anastrepha ludens* (Loew), data are rather controversial for *C. capitata*, *B. dorsalis*, and *Z. cucurbitae* [54]. Whether the cues exploited by males to aggregate in leks derives from their own pheromone emissions, or from the plants where leks occurs is still under debate. Indeed, in *C. capitata* it has been proposed that plant volatiles, rather than male-emitted pheromones, contribute to male aggregation in leks [62,63], with α-copaene, a natural sesquiterpene widely present in plants, being regarded as the primary male cue for lek formation [64,65,66,67]. Male pheromone has been shown to attract conspecific males thus acting as aggregation pheromone in some *Bactrocera* species [68,69,70,71,72,73]. Nonetheless, Kobayashi and colleagues reported no male-male attraction in *B. dorsalis* [74].

Interestingly, sex pheromones are not uniquely produced by males in all tephritid species. In the *Ceratitis*, *Anastrepha* and *Rhagoletis* genera, the volatile chemicals identified in the adult headspace are of male origin. Instead, the role of females in emitting volatiles is evident in the genus *Bactrocera*. In all *Bactrocera* species studied so far, both sexes produce and release pheromone chemicals, albeit interspecific differences exist. In *Bactrocera musae* (Tryon), a polyphagous pest distributed in Australia and Papua New Guinea mainland [75], both sexes produce volatile pheromones, but its complexity is higher in females than males [76].

In the olive fruit fly, *Bactrocera oleae* (Rossi), females release the pheromone that plays a central role in the mating system of this species [77,78,79,80]. However, earlier and most recent studies suggested that male-emitted chemicals elicit female attraction [81,82,83]. The finding that both sexes emit and perceive chemicals during courtship [50,84], together with the identification of male wing vibration in this species [50], encourage further research to better understand the role of chemical communication in the mating behaviour of *B. oleae*. So far, males have been regarded to swarm before settling on olive canopy [42]. The modality of male aggregation to form leks in this species needs to be further clarified too, in order to understand the relevance of chemical communication in a species where female-borne pheromones have been well documented both as a short- and long-distance cue [42]. Recently it was proven that ethyl decanoate emitted by olive fruit fly females attracts other females, and hence may be involved in female-female aggressive interactions on oviposition sites [85].

Courtship implies a series of ritualized actions, and it is much more complex in the genus *Ceratitis*, particularly in *C. capitata* [86], than in other tephritids, such as many species of *Anastrepha* [87,88] and *Bactrocera* [89]. However, recent studies are revealing that, even in species where it was believed to be simplified, such as in *Bactrocera tryoni* (Froggatt), courtship is more complex than previously thought [49]. During courtship, volatile chemicals are perceived by either one of both sexes using the antennae, while less volatile compounds, such as CHs, have been suggested to be important in later stages, when male and female touch each other during mating attempts and mating *per se* [35,42,90]. Indeed, in several tephritids, fore- and hind-leg interactions, as well as male foreleg interaction with the female abdomen, ‘kissing’ (i.e., touching of the labella), and male tapping with the labellum on female thorax have been reported [42,49,91]. For example, in *Ceratitis rosa* Karsch and *A. fraterculus*, sex-specific differences in the quantitative composition of CH profile, together with reported mating incompatibilities, further suggest the role of CHs as short-range semiochemicals [90,92,93].

Trophallaxis (i.e., female provision with gifts -oral, genital or transdermal- by males [91,94,95]), a common behaviour in tephritids, is considered as a courtship signal as well (see [96] for a review). While in *Anastrepha* species a pre-mating transfer of male oral products to females by labrum-to-labrum contact is well known [91,97,98], this phenomenon has been described in medfly only recently [96]. In this species, the consumption of male-produced oral droplets by the female appeared to increase her receptivity to mating, suggesting that the chemical composition of these droplets may be a tool to assess male quality [35,96]. Further research in the medfly and in other major tephritid fruit fly species is thus essential to unravel how these substances are perceived and how this behaviour fits in the complex semiochemical-based communication frame. Interestingly, a recent work demonstrates that *A. ludens* males regurgitate more than females, and propose that regurgitation and deposition of series of droplets organized in lines or spirals by some fruit fly species may play multiple functions (e.g., collecting bacteria from the environment, or eliminating ingested toxicants), including the production of oral pheromones [99]. Moreover, signalling males in some *Anastrepha* spp. deposit pheromone on the leaf surface by abdominal tip dipping [100]. Some of these deposited components persist on leaves up to one hour after removal of signalling males and are able to attract females [100]. Abdominal dipping with deposition of a viscous substance from the male cercus (i.e., the external appendage close to the digestive tract) has also been observed in *Rhagoletis boycei* Cresson [101].

After mating, tephritid females undergo an almost immediate switch from response to male pheromone to host plant-oriented olfactory behaviour to seek for appropriate oviposition background. This phenomenon has been well documented for the medfly at the behavioural level [102] and molecular data suggest that genes related to olfaction and/or foraging are also changing in their transcriptional profiles [103]. Mating-related differential expression in genes involved in chemosensory perception has also been detected in *Bactrocera* [104,105], and *Anastrepha* spp. [106], with females of *B. tryoni* showing mating-induced switches in olfactory preference [107].

The oviposition behaviour of tephritids is highly heterogeneous and display species-specific differences, such as daily patterns of oviposition, clutch size, patterns of positioning the ovipositor, duration of the oviposition bout, and preferred plant site for the oviposition [108]. In the medfly, oviposition behaviour has been widely studied and described as being structured in four steps: arrival to the fruit, exploring (i.e., survey of fruit surface with head, labellum and ovipositor), ovipositor puncturing and drawing following oviposition conclusion [109,110]). In the last phase, the fruit surface is again explored by the females, with the aculeus of the ovipositor protracted, and a HMP is deposited [111].

The presence of conspecific eggs and developing larvae in breeding substrates may dramatically alter the oviposition behaviour in phytophagous insects. In tephritids, females often mark already used hosts with a pheromone to avoid overexploitation of the specific resource and hence reduce/eliminate competition [112,113]. To maximise the chances of survival and success of their progeny, phytophagous insects tend to avoid egg laying in already explored host resources [29,108]. Host marking is particularly important in endophytic species, such as tephritids, in which females oviposit inside fruits or other plant tissues, with no visible damage and no emission of specific plant volatiles in response to infestation and presence of fruit fly eggs [114]. HMPs are generally applied by female drawing the ovipositor following an egg-laying event [112].

Mechano-, hygro- and gustatory receptors are located on female ovipositor in tephritids [115]. Gustatory sensilla present on the tarsi of *R. pomonella* exhibit sensitivity to HMP [116]. Sensilla types on female ovipositor have been described in several *Bactrocera* and *Zeugodacus* species (i.e., *Z. cucurbitae*, *Zeugodacus diaphorus* (Hendel), *B. dorsalis*, *Bactrocera minax* (Enderlein), *Zeugodacus scutellatus* (Hendel) and *Zeugodacus tau* (Walker) [117]; *B. tryoni* [118]), as well as in *R. pomonella* [119,120,121], but their characterisation is still patchy in species of the *Ceratitis* and *Anastrepha* genera [122].

Interestingly, HMPs have been shown to either deter or enhance oviposition, depending on concentration and other factors [123]. Deterrent effects induce different behaviours in responding females including suppression/disruption of oviposition, reduction of the number of egg clutches per fruit and of egg number per clutch, and dispersion to less infested (occupied) areas [108,114,124,125,126,127,128]. Although HMPs are predominantly recognised by individuals of the same species [29,129], interspecific perception of HMPs has also been described in tephritids. Cross-recognition has been demonstrated among species of the *Rhagoletis* [130,131], *Anastrepha* [132] and *Ceratitis* genera [33,34]. The host-marking behaviour may display different features even among species of the same genus. For example, small-sized fruit specialists (e.g., *Rhagoletis alternata* (Fallen), *Rhagoletis indifferens* Curran, *R.* *pomonella* and *R. cerasi*) often deposit HMPs [133]. Conversely, members of the *Rhagoletis* *suavis* group rarely mark the host targets [134,135] and commonly tend to lay eggs on infested fruits [134,136,137]. Two hypotheses have been proposed for the sporadic host-marking behaviour of the *suavis* group. According to the “no HMP deposition” hypothesis, all species of this group use as host walnut species (*Juglans* spp.), which are not infested by other flies of the genus in North America, and hence provide a competition-free resource for larvae [133]. The second hypothesis proposes the occurrence of a “male host-marking behaviour”. *Rhagoletis boycei* males indeed usually touch the host fruit depositing a substance on its surface and females preferentially oviposit on the unmarked fruit [101]. According to this hypothesis, male host-mark replaces the female’s one, causing a reduction in female marking behaviour. Male host-marking behaviour has been described in two species of the *suavis* group, *R. boycei* and *R. suavis* (Loew) [113].

The drawing of the aculeus after oviposition without an evident release of HMP has been described in *B. dorsalis* [138], *B. tryoni* and *B. jarvisi* (Tryon) [139], and *Z. cucurbitae* [140]. Instead of using their ovipositor right after oviposition, olive fly females spread the olive juice that leaks from the oviposition wound over the fruit surface using their labella. This behaviour appears to prevent other females from ovipositing on the same fruit [141,142,143].

## 3. Volatile Pheromones

### 3.1. Tissues Involved in Volatile Pheromone Production

Male pheromones seem to be released primarily from glands positioned in the rectum in *Ceratitis* [144,145], *Bactrocera* [74,79,146,147,148], and *Anastrepha* spp. [149,150,151]. However, interesting variations have been reported. This is the case of the goldenrod gall tephritid fly *Eurosta solidaginis* (Fitch), which possess no anal glands but enlarged rectum and pleural epidermis that have been suggested to be involved in pheromone production and/or storage [152].

In general, the rectal valve of *A. fraterculus* and *C. capitata* is located at the distal portion of the colon, while the intestinal canal enlarges in a chamber comprising four rectal papillae projecting in the lumen of the rectal ampulla (i.e., the anterior rectum) [153]. In its proximal region, the ampulla is lined with epithelial cells that increase in number and are organized in folds in the distal portion. In several *Anastrepha* species, the structure of the female rectum is similar, with differences in the features of the epithelium [154]. In *Bactrocera* spp. (e.g., *B. oleae*, *B. dorsalis* and *B. tryoni*), the rectal glands of males and females display a remarkable sexual dimorphism [155,156,157]: muscles surrounding male glands are more abundant, suggesting a more complex contraction capacity to support the pheromone-storage and -release functions. The rectal sac (i.e., an evagination in the rectal gland) is present only in males. Because they are not surrounded by muscles and directly exposed to the haemolymph, the rectal pads, which extend into the gland as rectal papillae, have been suggested to be involved in transporting chemicals into the rectal gland [157].

During sexual calling, the anal tube is protruded to allow the epithelium of the distal rectum to be extruded. In medfly, when everted, the folded rectal epithelium is expanded in a balloon-like structure [144]. This evagination of the anal membranes at the abdominal tip, which appears as a droplet, has been described in *Anastrepha* and *Ceratitis* species, and it has been explained as a mean of expanding the evaporative surface, thus increasing pheromone emission and, consequently, attractiveness [100,150,158]. In addition, this behaviour is accompanied by protrusion of male expansion of the pleural abdominal region generating two lateral blisters [149,159].

Males of *C. capitata* and *C. rosa* display three types of sex-specific glands, *(i)* the anal glands, *(ii)* the pleural glands and *(iii)* the dimorphic salivary glands [160]. The pair of anal glands open onto the external cuticle close to the anal opening [160,161]. Males of *Anastrepha* species display only the pleural glands and dimorphic salivary glands [151,160]. In *Bactrocera* species (e.g., *B. oleae*, *B. tryoni* and *B. dorsalis*), both the anal glands and the dimorphic salivary glands are absent [146,160,162]. Currently, detailed information on *Rhagoletis* species are lacking, although the early work of Nation [160] found neither dimorphic salivary glands nor pleural glands in both *R. pomonella* and *R. juglandis* (Cresson).

**Figure 3:**
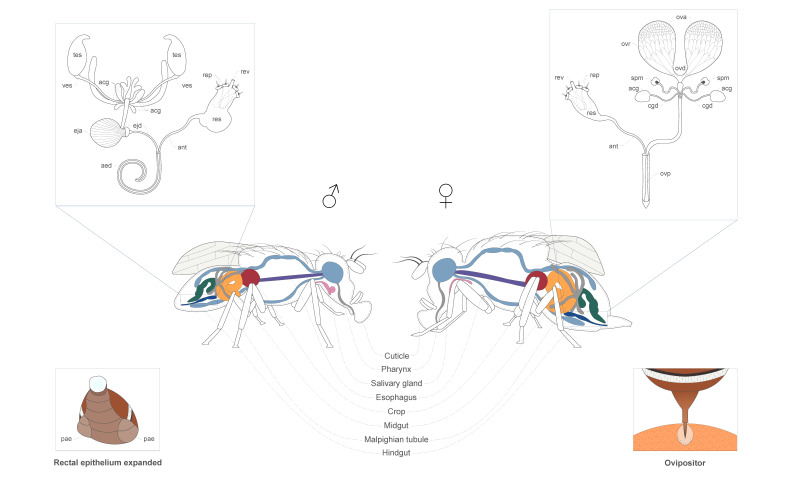
Tissues involved in semiochemicals’ production in tephritids. Diagrammatic representation of a male (left) and female (right) generalized tephritid showing the tissues/body compartments involved in pheromone production. Reproductive tracts and rectum are shown in upper left (male) and right (female) boxes. Bottom left and right boxes show the abdomen of a calling male, showing the expanded rectal epithelium (pae, pleural abdominal expansion), and the ovipositor of a female. Abbreviations: Male reproductive system: tes (testes), ves (vas deferens), acg (accessory glands), ejd (ejaculatory duct), eja (ejaculatory apodeme), aed (aedeagus). Female reproductive system: ova (ovaries), ovr (ovariole), ovd (oviduct), spm (spermathecae), acg (accessory glands), cgd (colleterial gland duct), ovp (ovipositor). Rectum: rev (rectal valve), rep (rectal papilla), res (reservoir containing pheromone), ant (anal tube).

In addition to the key function played by rectal tissues, pheromones have been later found to be also released orally. Salivary glands release pheromone components in *Anastrepha* spp. [149,150,151,163,164]. These glands are sexually dimorphic, with male salivary glands being ball-like structures associated with the crop [99,160,165]. Salivary glands have been suggested to be involved in the storage and, potentially, synthesis of pheromone components in medfly males [166]. This is supported by the fact that some chemicals of the pheromone blend identified in medfly male headspace were also detected in the extracts of salivary glands. Figure 3 summarizes the tissues involved in pheromone production in tephritids.

### 3.2. Composition of Volatile Pheromones

In tephritids, the volatile pheromone is a mixture of diverse chemical compounds with different isomers [167]. These chemicals are either newly synthesised or produced from precursors acquired from the diet. Plant-borne chemicals introduced with the diet become integrated into the pheromone mixture and contribute to male mating success. Diet-derived chemicals can unmodified be incorporated into the pheromone blend (e.g., raspberry ketone (RK) in *Z. cucurbitae* [168,169]) or can go through conversion in other compounds that are then used in the pheromone (e.g., ME in *B. dorsalis* that is converted in 2-allyl-4,5-dimethoxyphenol and *trans*-coniferyl alcohol [68,69,170,171]). *Bactrocera dorsalis* males are attracted to ME, and feeding on this compound was shown to directly benefit mating success [171,172].

The pheromone mixture comprises both major, minor and trace compounds, with the complete blend displaying stronger effects than individual compounds or a mix of a subset of compounds. Indeed, medfly females respond differentially to mixtures of major male pheromone compounds than to the complete blend [173]. In *B. oleae*, olean (1,7-dioxaspiro[5.5]undecane) is the major component of female sex pheromone, and it is more attractive to males than the other identified components (e.g., α-pinene, nonanal, and ethyl dodecanoate) [174]. However, the combination of all chemicals is more attractive than olean alone (see [171] for a review).

The composition of the pheromone mixture has been investigated using two main approaches: (i) sampling the headspace of calling males (or females in the case of Bactrocera species), (ii) extracting the chemicals from the rectal glands. Volatiles captured in the headspace have been so far collected and characterised in 18 tephritid species (Table 1; Table S1), while gland extracts have been obtained from 26 species (Table 2).

The two most represented chemical classes of the volatile compounds captured in the headspace in *Anastrepha*, *Bactrocera, Ceratitis*, *Rhagoletis* and *Zeugodacus* species are fatty acyls and organooxygen compounds. Prenol lipids, which represent most compounds in *Ceratitis* and *Anastrepha* species, and are abundant also in *Rhagoletis*, are instead poorly represented in *Bactrocera* and absent in the emissions of *Z. cucurbitae*. Lactones are particularly abundant in *Anastrepha* species (Table S1).

**Figure 4:**
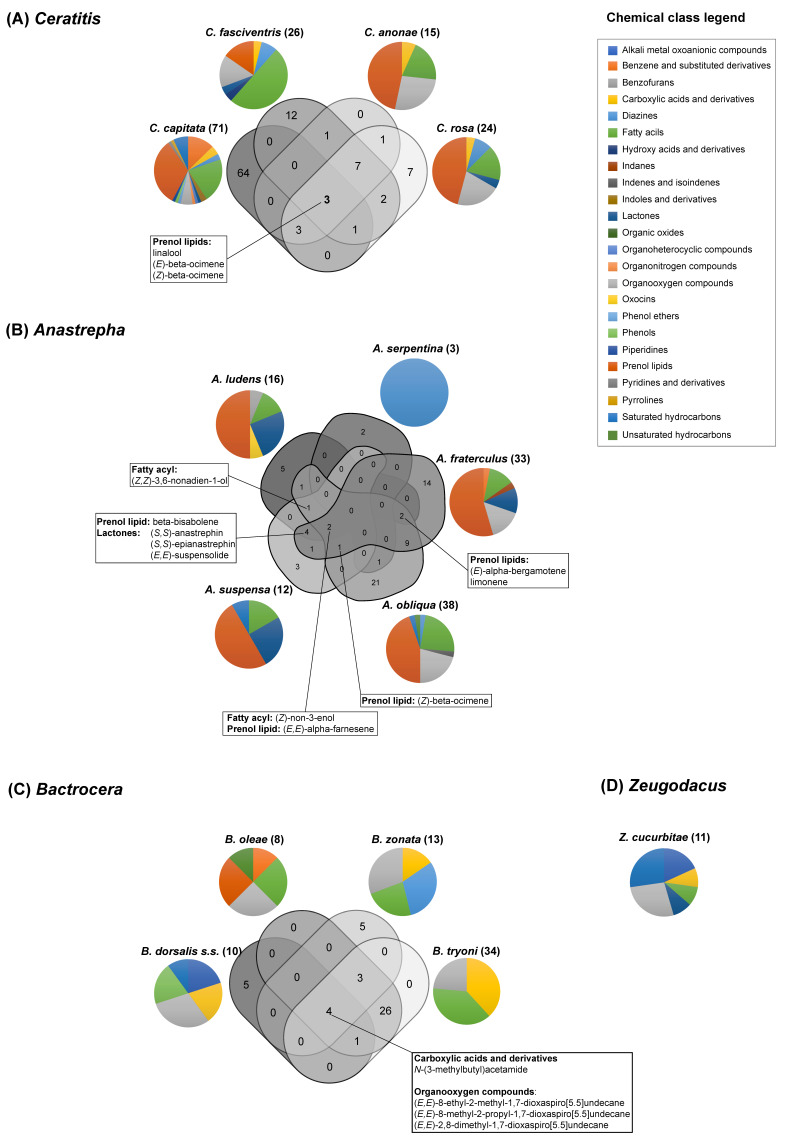
Overlap among chemicals identified in the headspace emissions of tephritids. Venn diagrams (http://bioinformatics.psb.ugent.be/webtools/Venn/) (accessed on 10 March 2021) illustrating the number of common and unique chemicals found among the compared species in the (**A**) *Ceratitis*, (**B**) *Anastrepha*, and (**C**) *Bactrocera* genera. (**D**) For the genus *Zeugodacus*, the number of compounds emitted by *Z. cucurbitae* are reported. Pie charts represent the proportion in chemical classes of the total number of compounds (in parenthesis) isolated for each species.

Within each genus, it is evident that only few compounds are shared among species (Figure 4). For example, in all four *Ceratitis* species investigated so far, namely *C. capitata*, *Ceratitis fasciventris* (Bezzi), *Ceratitis anonae* (Graham), and *C. rosa,* only three volatile chemicals, all belonging to the prenol lipid class, are emitted by males. These three compounds are linalool, (*E*)-β-ocimene, and (*Z*)-β-ocimene that also occur naturally in the host plants. In *C. capitata*, the perception of plant volatiles has been investigated by electroantennogram (EAG) and behavioural studies demonstrating that linalool, a compound representative of immature citrus fruit associated with high toxicity against immature stages of fruit flies and considered as an important compound conferring resistance against fruit fly larval development, has a significant deterrent effect [240]. Linalool was reported by numerous studies as an active constituent of medfly male sex pheromone that elicits a strong EAG response [218,221,241]. It also triggers antennal depolarisation in females of *C. fasciventris*, *C. anonae* and *C. rosa* (so-called *Ceratitis* FAR complex) [214].

In *Anastrepha*, (*Z*)-non-3-en-1-ol (a member of the fatty acyl compound class) is the unique compound shared among *A. fraterculus*, *A. ludens*, *A. obliqua*, and *A. suspensa*. This compound is a typical host plant component and has been shown to elicit an active behavioural response in all four species [175,188,193,242,243]. For example, *A. fraterculus* females are attracted to (*Z*)-non-3-en-1-ol [175]. These behaviours may be interpreted as a first step in the complex mating process of this species, i.e., attracting females to the mating site. Since mating is strongly associated with host plants, the use of plant typical compounds (e.g., limonene and pinene, among others) would help females to find simultaneously mating and oviposition sites [244].

*N*-(3-methylbutyl)acetamide (carboxylic acids and derivatives class) and the three organooxygen compounds (*E*,*E*)-8-ethyl-2-methyl-1,7-dioxaspiro[5.5]undecane, (*E*,*E*)-8-methyl-2-propyl-l,7-dioxaspiro[5.5]undecane, and (*E*,*E*)-2,8-dimethyl-l,7-dioxaspiro[5.5]undecane are shared among *B. dorsalis s.s.*, *B. oleae*, *B. zonata* (Saunders) and *B. tryoni*. Interestingly, *N*-(3-methylbutyl)acetamide elicits female attraction in *B. dorsalis* and *B. carambolae*, but also in *Z. cucurbitae* [72,204,229]. The spiroacetal (*E,E*)-2,8-dimethyl-1,7-dioxaspiro[5.5]undecane elicits an antennal response in *B. musae* males, suggesting a biological role for this compound [76].

It is noteworthy that research efforts in investigating pheromone composition have been particularly intense in certain species such as the medfly, and this may reflect in the number of identified compounds. Future studies integrating multiple analytical approaches in all species will enable a more extensive description of the shared and unique chemical signatures of tephritid volatile pheromones.

The differences in the male pheromone composition that have been reported may be partially linked to technical aspects (e.g., different sampling techniques, experimental variables such as airflow, sampling duration, and column features), as well as to several factors affecting pheromone production and release, such as the time of the day [149,200], social context [149], food availability [199], and diet [220,245]. Larval diet (i.e., the different host fruits utilised) and adult age affect both the total amount of pheromone emitted by males, the relative quantity of major components, and the presence of specific minor compounds in the medfly [220]. Similarly, in *A. ludens*, the profile of the male pheromone blend and the relative abundance of the chemical components change in response to different host fruits used for larval development [245]. Moreover, qualitative and quantitative differences in the composition of male-emitted volatile pheromone have been detected among laboratory strains and different populations in *A. fraterculus* [176,177], and among laboratory strains and wild populations in *C. capitata* [221]. In addition, emission of male pheromone increases in *Anastrepha* species in response to treatments with methoprene (a synthetic analogue of the juvenile hormone), with important implications in gaining deeper insights into tephritid reproductive physiology and in enhancing the application of management methods such as the release of sterilised males [246,247].

The components of male pheromone also display different volatilities [248]. Six alkanes and related compounds have been identified in the headspace of medfly males reared on a standard wheat bran-based larval diet [220]. These compounds display poor volatility and do not belong to the published CHs identified in the cuticle of adult medflies. Thus, similar to *Drosophila* species [249,250], medfly seems to be able to deposit on the substrate and emit in the surrounding air CH-like compounds, which may serve as short/medium distance cues for mate localisation. The ability of males to deposit pheromone on the substrate, in addition to aerial pheromone release, has also been reported in *A. suspensa* [63,100]. Moreover*,* saturated C25, C27 and C29 hydrocarbons have been detected in the volatile pheromone of male melon fly *Z. cucurbitae* [205].

### 3.3. Does Host-Preference Affect the Volatile Pheromone Bouquet?

Pheromone precursors are acquired from four main sources: (i) *de novo* synthesis, (ii) conversion of precursors that insects acquire from host plants or substrate, (iii) direct incorporation from the host plants, and/or (iv) from endosymbionts [21,251,252,253,254]. Thus, it is likely that different variables influence the pheromone blend.

The chemical classes of their pheromone components and their specific identity are more similar among species of *Anastrepha, Ceratitis* and *Rhagoletis* genera, and rather different from species of the genus *Bactrocera*. The monophagous *B. oleae* is the most diverse species among the genus, with the majority of its pheromone volatiles being species-specific. To elucidate whether the composition of the pheromone components is related to feeding strategies of different species, we analysed the lists of volatile compounds identified in all species of the genera *Anastrepha, Ceratitis*, *Bactrocera* and *Zeugodacus*. This analysis could not be extended to *Rhagoletis* genus, as only the volatiles emitted by *R. cerasi* have been identified [226] so far, and a single compound has been isolated in *R. batava* [61].

**Figure 5:**
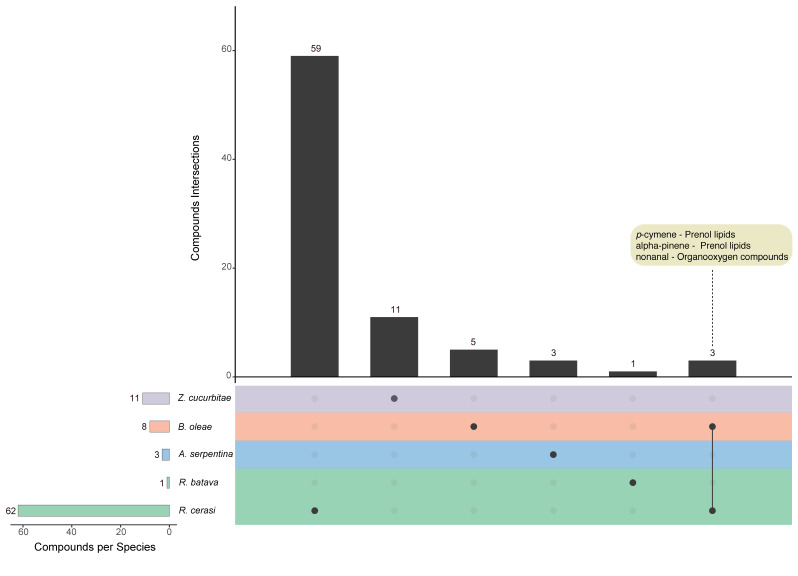
Comparison among the chemicals identified in the headspace emissions of monophagous and oligophagous tephritid species. UpSet plot showing unique and overlapping chemicals across five species belonging to the *Zeugodacus*, *Bactrocera*, *Anastrepha* and *Rhagoletis* genera. The intersection matrix is sorted in descending order. Connected dots represent intersections of overlapping chemicals and horizontal bars show the total number of compounds identified in each species headspace. The plot was generated using the UpSetR package in R [255].

The species with a monophagous or oligophagous feeding strategy mostly emit chemicals that are not shared with other species with the only exception of nonanal, α-pinene and *p*-cymene, which are in common between *B. oleae* and *R. cerasi*. Figure 5 shows the number of chemicals identified in each species as well as the intersections of overlapping compounds, represented by connected dots (see Table S2 for the complete lists of unique and overlapping chemicals).

**Figure 6:**
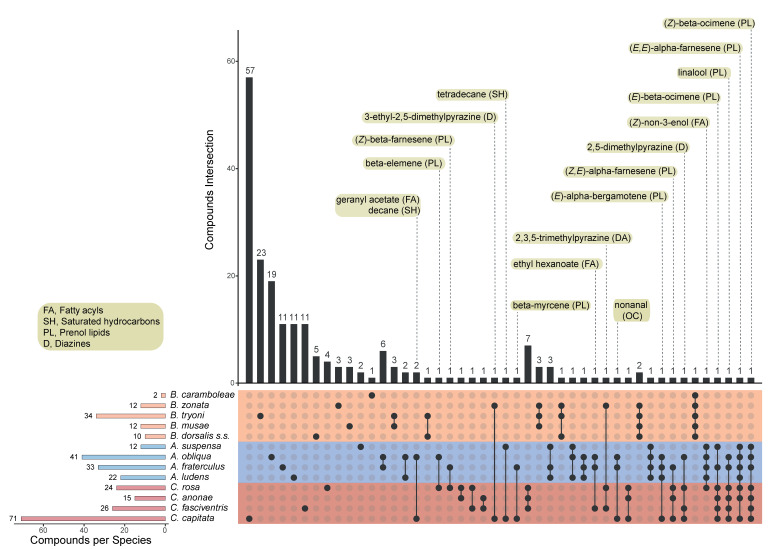
Comparison among the chemicals identified in the headspace emissions of polyphagous tephritid species. UpSet plot showing unique and overlapping chemicals across five species belonging to the *Bactrocera*, *Anastrepha* and *Ceratitis* genera. The intersection matrix is sorted in descending order. Different genera are indicated in different colors. Connected dots represent intersections of overlapping chemicals and horizontal bars show the total number of compounds identified in each species headspace. The plot was generated using the UpSetR package in R [255].

The polyphagous species shared more compounds, as shown by the higher number of connected dots in Figure 6 (see Table S2 for the complete lists of unique and overlapping chemicals). This finding is particularly evident between species belonging to the same genus.

Figure 6 also reports the identity of the chemicals that are shared between different genera. It generally appears that the members of the *Anastrepha* and *Ceratitis* genera share a higher number of compounds, with chemicals belonging to the prenol lipids class being most frequently in common. Conversely, *Bactrocera* species display quite unique pheromone blend features. The only two compounds to be shared belong to the diazine class and are in common with *Ceratitis* species. This suggests that at least two major evolutionary forces, i.e., genomic background and host feeding strategy, interacting in a complex manner, have shaped the pheromone blend in these species. A comprehensive molecular phylogeny of tephritid species will be of great help in disentangling the effects of genome evolution and environmental selective pressures in shaping semiochemical-based behaviour in these species. Further research is required to deeply investigate how feeding strategies affect the production of semiochemicals, also with respect to the capacity of colonizing new hosts, a feature that characterises several invasive tephritid pests. A strong effect of larval host on the pheromone bouquet produced by the polyphagous species *A. ludens* and *A. obliqua* has been recently demonstrated [245].

### 3.4. Analytical Approaches to Unravel Pheromone Composition

The complexity of the signals involved in tephritid chemical communication, together with their presence in small amounts in natural systems, stimulated the adoption of very sensitive high-resolution analytical methods for their detection, identification and testing. These strategies include both conventional as well as reverse chemical ecology approaches. The workflow of conventional chemical ecology (CCE) approaches generally involves the following steps: (i) preliminary observation of the existence of pheromone-mediated communication in the target species, (ii) volatile pheromone sampling, (iii) characterisation of pheromone blends through analytical techniques, (iv) chemical synthesis of the identified compounds, (v) evaluation of their electrophysiological activity, (vi) behavioural assays (bioassays) to test the role of the isolated semiochemicals in laboratory and/or field set-ups in order to confirm their pheromone identity [256]. Recently, a different approach began to be utilised for the discovery of semiochemicals, reverse chemical ecology (RCE) [257]. Similarly to what occurs for receptor-based drug discovery, RCE exploits odorant binding proteins (OBPs) as molecular targets for the screening of behaviourally-active chemicals based on their binding affinity [258]. The workflow of RCE comprises the following steps: (i) identification of OBP targets through genomics and bioinformatics approaches, (ii) characterisation of OBP expression profile, (iii) purification and 3D-structural analysis, (iv) in vitro OBP: ligand binding assays using semiochemicals collected from insects or pure compounds, (v) in silico selection of test ligands, (vi) bioassays to verify the effects of the identified semiochemicals on insects *in vivo*. The key aspects of these approaches are described in the following sections.

#### 3.4.1. Conventional Chemical Ecology Approaches

The components of the complex blends of tephritid pheromones can be isolated, identified and tested using interdisciplinary approaches involving bioassays, sensory physiology, analytical and organic chemistry, and biochemistry. Recent breakthroughs in analytical techniques allow the rapid screening of semiochemicals with more sensitive bioassays and their isolation and identification from relatively smaller amounts of material. Several non-destructive and artefact-free methods are available for collecting natural odorants from living organisms. Examples include the adsorption of odours on different polymer matrices contained in cartridges or filters. Trapped odours can be desorbed thermally or eluted with organic solvents followed by analysis using gas chromatography. The direct coupling of a chemical (flame ionization detector and/or mass spectrometer) and biological detector (e.g., the use of an insect antenna) permits simultaneous isolation and identification of bioactive components from trapped odours. Furthermore, the discovery of more efficient chemical synthetic methods now allows the state-of-art synthesis of semiochemicals of high purity whose field activity may provide answers to ecological and evolutionary questions associated with the importance of the chemical in the behaviour of the target insect [259].

##### Volatile Collection and Analytical Techniques for Their Identification

Chemical identification of tephritid volatiles requires a chromatographic separation followed by detection using spectrometric analytical methods. Although a wide range of methods is available in principle, the number which is suitable in practice depends upon the amount of insect material that can be obtained.

Solvent extraction of tephritid rectal glands using standard solvents such as heptane, hexane, methanol, ethanol, dichloromethane and ethyl ether is commonly used [166,213,231,232]. More recently, mixtures of solvents (acetonitrile/water and methanol/acetonitrile/water) were applied for the extraction of semiochemicals from the whole body of adult medflies [260].

Solid phase microextraction (SPME) is a solvent free, pre-concentration technique developed by Arthur and Pawliszyn [261] for application in solid, liquid, or gaseous samples (reviewed in [262]). The results obtained using SPME fibres are similar to those obtained with solvent extraction [263,264,265]. SPME is a known and effective alternative to liquid-liquid extraction. It provides some advantages over liquid-liquid extraction process because of reduced solvent consumption. In tephritids, different SPME coating materials have been used for volatile collections. A polydimethylsiloxane/divinylbenzene fibre (PDMS/DVB) was applied for pre-concentration of volatile compounds emitted by male and female medflies in different mating status (virgin or mated), and age (3 or 9 days old) [222]. In total, 70 compounds of diverse chemical classes such as alcohols, acids, aldehydes, terpenes, branched hydrocarbons and esters were reported and identified by gas chromatography coupled with mass spectrometric detection (GC-MS) [222]. Similarly, PDMS fibres have been used to compare the composition of the pheromone of medfly males from a standard laboratory strain reared as larvae on laboratory media and fresh fruits [220]. Five and 30 day-old males have been used, with 36 and 27 chemicals (mostly belonging to terpenes, amides, esters and alkanes) identified to be emitted by these two age-classes, respectively [220]. In a recent work on *C. capitata* volatiles, divinylbenzene/carboxen/polydimethylsiloxane (DVB/CAR/PDMS) combined with GC-MS and gas chromatography-flame ionization detection (GC-FID) techniques resulted in the identification of 27, 23 and 29 compounds from larvae, pupae and adults, respectively [266]. PDMS/DVB SPME fibres have been applied to collect volatiles produced by *B. zonata* males and females and *R. batava* males, respectively [61,213].

Dynamic headspace is a common method for the collection of volatiles produced by fruit flies. The volatiles emitted by virgin-calling flies are collected using a modified technique of an air-collection apparatus as described by Nation [149]. In this technique, a purified airstream is blown over living flies enclosed in a glass chamber. The volatiles are collected onto traps containing adsorbents such as SuperQ, Tenax, Activated Carbon, etc. [213,221,223]. The air flow directed through the apparatus is controlled by flowmeter. Volatile collections are usually performed for 24 h. Afterwards, the traps are washed with heptane, hexane, diethyl ether or ethanol and the obtained extract is analysed by GC-MS, electroantennography, gas chromatography coupled to electroantennographic detection (GC-EAD) methods and used for bioassays.

GC-MS is one of the most useful tools for chemical analysis of volatile semiochemicals. The gas chromatography provides high-resolution separation of components within a complex mixture, and the mass spectrometric detection supplies structural information in addition to its role as a sensitive detector (the current limit of detection is at femtomole levels). By selection of the appropriate capillary column, practically all volatile organic compounds can be separated, including carboxylic acids, ketones, aldehydes, alcohols, aromatic compounds, and hydrocarbons [180,215,226,267,268,269,270].

For identification of the absolute configuration of tephritid volatile semiochemicals, the chiral column or chiral GC-MS can be used, as recently applied for pheromone identification of *R. batava* [61]. Most commercial chiral GC phases currently available are composed of modified cyclodextrins, which give a wide range of enantiomeric separations, but have the disadvantage of being thermally unstable above 200 °C, and are therefore useful for relatively volatile compounds only. Like chiral nuclear magnetic resonance (NMR) studies, chiral GC requires homochiral or enantioenriched synthetic standards, but unlike the NMR technique, only nanograms of impure material are necessary [271]. Two-dimensional gas chromatography with time-of-flight mass spectrometric detection (GC × GC-TOFMS) is a recently developed analytical technique that offers a solution to the chromatographic co-elution and provides high sensitivity and selectivity. In principle, the method consists of two GC systems (GC × GC) equipped with columns of different polarity connected by an interface with an integrated cryogenic trap. The cryogenic trap repeatedly condenses compounds eluting from the primary column and releases them periodically as short pulses to the secondary column. Parameters like duration and frequency of both condensation and injection pulses are variable and allow precise tuning of the instrument according to the requirements of the analysis. Since the GC × GC produces very narrow peaks (down to 50 ms, depending on the frequency of the cryogenic modulation) a time-of-flight mass spectrometric detector (TOFMS) with a high acquisition rate (up to 500 spectra per second) is required. The pulsed nature of the TOFMS source of ionisation further enhances the system accuracy by avoiding the spectral skewing common in a continuous ionisation mode. GC × GC with TOFMS detection thus operates with a high precision independent of the concentration range [272,273]. This method has been applied for analyses of fruit fly semiochemicals produced by species of the genera *Anastrepha*, *Bactrocera* and *Ceratitis* [177,214,221,274].

Gas chromatography coupled with Fourier transformed infrared spectroscopy (GC-FTIR) is relatively sensitive (detection threshold 10–100 mg) and particularly useful for identifying geometrical and positional isomers or functional groups. Thus GC-FTIR is an ideal instrument for the study of volatile organic compounds. Medfly synthetic attractants composed of *trans*-trimedlure isomers and *cis*-trimedlure isomers were analysed by GC-FTIR spectroscopy [275].

Matrix-assisted laser desorption/ionization with time-of-flight mass spectrometry (MALDI-TOFMS) and desorption electrospray ionization (DESI) mass spectrometry are soft ionization techniques developed for the analysis of biomolecules (biopolymers such as proteins, peptides and sugars) and large organic molecules (such as polymers, dendrimers and other macromolecules). However, they have been applied very sporadically for studies of the fruit fly pheromone components [276].

High performance liquid chromatography (HPLC) coupled to a MS detector is at present the favoured technique for the study of polar non-volatile tephritid semiochemicals. Other types of detectors that are commonly used with HPLC separation are UV and ELSD (evaporative light scattering) detectors [277,278,279,280]. Normal phase and reversed phase HPLC separation are still popular in lipid analysis. The mobile phases usually consist of methanol, propan-2-ol, or *n*-hexane. Chromatographic columns with C18-phases still prevail. A few separations were reported on C8-columns. In a recent metabolomic study of *B. dorsalis* larvae, liquid chromatography-mass spectrometry (LC-MS) and GC-MS were applied for the characterisation of endogenous metabolite changes and biochemical effects of azadirachtin [281].

NMR spectroscopy is one of the most informative, but least sensitive, modern spectrometric methods. Fourier transform ^1^H and ^13^C NMR spectroscopy require approximately 100 μg and 10 mg pure material, respectively (50 ng – 1 μg with very expensive NMR-nanoprobe). Since many insect pheromones are only present in nanogram amounts, a very high number of individuals are required. NMR is widely used to determine the absolute configuration of optically active compounds (semiochemicals), either using chiral shift reagents, or by converting enantiomers to diastereoisomers prior to spectroscopic study, or using chiral solvent. The use of NMR for absolute identification of semiochemicals has two main disadvantages: isolation of the pure substance is required, and an enantioenriched or a homochiral synthetic standard is needed. In modern semiochemical research, NMR spectroscopy is normally used only when insufficient structural information is provided by more sensitive methods [282]. Baker and Heath [283] applied NMR spectroscopy for the identification of lactone pheromone components emitted by *A. suspensa* and *A. ludens.*

##### Chemical Synthesis

The chemical synthesis of tephritid pheromones is a key step towards producing pheromone compounds for use in bioassays (usually very low amounts), and for insect pest monitoring and management purposes. Moreover, their absolute configuration is an important determinant of their biological activities [284]. In 1973, Jacobson and colleagues isolated and synthetized the first medfly pheromone components, i.e., methyl (*E*)-non-6-enoate and (*E*)-non-6-en-1-ol that were attractive eliciting also sexually excitatory response to females in laboratory trials [215]. Three additional major components of the medfly pheromone, namely ethyl (*E*)-oct-3-enoate, geranyl acetate, and (*E*,*E*)-α-farnesene were isolated and synthetized later [225]. In 2010, Olszewski and Grison [285] reported a novel and versatile synthetic approach to the preparation of (*E*)-non-6-en-1-ol.

Since the first successful studies on the isolation and synthesis of the medfly pheromone components, several efforts have been made to characterise the volatiles emitted by *Bactrocera* and *Rhagoletis* species, mainly using the enantioselective approach. Moreover, in the case of chiral pheromones, which occur in tephritids, the synthesis and following bioassays using stereoisomers are essential for recognition of the stereochemistry-bioactivity relationships. This knowledge also has practical implications for pest control as in most chiral pheromones only one enantiomer is biologically active [284]. In the case of *B. oleae*, olean (1,7-dioxaspiro[5.5]undecane) was isolated and identified as the major component of the female-produced sex pheromone in 1980 [79]. (4R*,6R*)-4-hydroxyoleane and (3R*,6R*)-3-hydroxyoleane (the asterisks indicate the chiral centers) were then isolated as minor components [79]. Bioassays involving the synthetic enantiomers of oleane showed that (*R*)-oleane is active on males, while (*S*)-oleane on females, and GC analyses showed that females can produce (±)-oleane [207]. The synthesis of enantiomers of 4-hydroxyoleanes was conducted by Mori and co-workers [286] and a recent study identified and synthesised 11 new *B. oleae* female-specific components [287].

Knowledge of pheromone composition in *Rhagoletis* is still limited to *R. cerasi* and *R. batava*. In this last species, Buda and colleagues recently applied GC-MS analyses of fly headspace and found that males emit (-)-δ-heptalactone. The authors then synthesised the two enantiomers of (-)-δ-heptalactone using enantioselective synthesis and found that only (-)-δ-heptalactone elicited an electrophysiological response in both sexes, proposing that this chemical may act as aggregation pheromone [61].

Despite these research efforts, similar studies focusing on the optimisation of chemical synthesis of pheromone components are completely missing for the genus *Anastrepha*, as well as for many other species belonging to the *Bactrocera, Ceratitis*, *Rhagoletis* and *Zeugodacus* genera.

##### Identification of Electrophysiologically-Active Volatiles

GC-EAD uses the electrophysiological response from a dissected insect antenna to assign activity to a gas chromatographic peak. The GC eluent is normally split between a flame ionization detector (FID) and the insect antenna, which is connected to an amplifier and recorded by silver or platinum electrodes. The response from each detector is recorded simultaneously so that an EAD response can be correlated with a specific peak in the FID chromatogram. This technique is particularly useful for the assignment of activity to chemicals which may be present as minor components in complex mixtures, although it provides no information about the function of the active compounds. GC-EAD analyses of male pheromone or rectal glands extracts have been performed on several tephritid species, including *C.* *fasciventris*, *C.* *anonae*  and  *C. rosa* [214], *C. capitata* [221,241], *B. oleae* [288], *A. serpentina* [193]*, A. fraterculus* [177,289,290,291], *A. obliqua* [243] and *A. striata* [292]. Additional studies performed GC-EAD analyses on the headspace volatiles of both sexes, in the species *R. batava* [61], *Bactrocera frauenfeldi* (Schiner) [293], *B. musae* [76]*,* and *C. capitata* [223]. In addition to GC-EAD*,* gas chromatography-electropalpogram detection (GC-EPD) has begun to be used to test the responses of tephritid maxillary palps to the pheromone emissions [293].

Furthermore, electroantennography-based experiments can be performed independently from gas chromatography. In this case, pure compounds are employed to assess the response to a certain stimulus and the required dose to elicit a detectable response. In this case, chemicals are delivered to insect antennae/palps in controlled conditions by pumping the odour from a reservoir [256]. Since early works in the ’80s [244,294,295], numerous EAG-based studies have been performed in tephritids to test the electrophysiological response to volatiles of fruits and flowers [296,297,298,299], artificial attractants [300,301,302,303,304], as well as pheromone components [305,306]. The use of GC-EAD brought the EAG-based approaches to a higher level of sophistication by using the antenna/palp of the target insect as a detector for the gas chromatograph. However, while electroantennography is an excellent technique to quickly measures the change in the electrical potential between distal and proximal sections of the antenna/palp provoked by olfactory stimulation, EAG amplitude depends on the position of the electrode, the strength of the connection, and insect vitality [305,306]. Thus, EAG is considered a qualitative indicator of olfactory reception [307]. Also in tephritids, EAG signals are indeed known to vary in relation to the relative density of sensilla, which display a specific distribution on the funiculus, at the electrode location [308,309]. To increase the recording specificity, a novel method was developed integrating EAG recordings at multiple antennal positions with current source density (CSD) modelling [310,311] useful to map the functional activation of individual antennae. This method was applied to six tephritid species, i.e., *C. capitata*, *C. catoirii* (Bezzi), *Neoceratitis cyanescens* (Bezzi), *B. zonata*, *Z. cucurbitae*, and *Dacus demmerezi* (Bezzi) to measure the response to seven volatile chemicals emitted by fruits and plants at four position in the funiculus.

A more quantitative measurement of the olfactory response, although requiring significant experimental effort on multiple individuals, can be achieved using single sensillum recording (SSR). SSR allows to measure the action potentials generated by olfactory sensory neurons (OSNs) within individual sensilla on antennae or palps using an electrode in contact with the lymph of the extracellular receptor [307]. To the best of our knowledge, this approach has been used only in two tephritids so far, but involving, in both cases, fruit odours as stimuli. The sensilla located on the tarsi of *C. capitata* [312], and the antennae of *R. pomonella* [313] were investigated.

##### Behavioural Assays to Identify Active Compounds

Bioassays play key roles in the study of tephritid semiochemicals [271]. Bioassays allow to assess the behavioural effect(s) (e.g., lek formation, male signalling, courtship, copulation, host finding for oviposition, HMP deposition) of synthesised active compounds (pure or in mixture) with respect to host or mate interactions, differently from EAG assays that provide the electrophysiological responses of isolated organs, structures or olfactory receptors (ORs). The biological response measured by a bioassay is essential to attribute to a molecule or a mixture of chemicals the pheromone identity. Behavioural effects in tephritid species can be evaluated in laboratory, semi-field and field conditions where both the stimuli and the background in which the stimuli are presented are tightly controlled [314]. Techniques for insect bioassays have been widely reviewed (see for example [256,315,316,317]) and widely applied to tephritid species, for which most experiments have been so far performed in laboratory conditions. Initial studies were done in close-range cage bioassays estimating medfly female attraction to male pheromone components based on landings on filter paper soaked with olfactory stimuli [218]. Currently used setups adopted for the study of tephritid olfaction include (i) two-choice systems, (ii) flight tunnels, and (iii) multi-arm olfactometers. Two-choice systems, using arenas (i.e., observation chambers) [174,318,319], as well as Y-tubes/T-maze [85,231,293,320], have been used to evaluate attractive or repulsive responses following exposure to pheromone (isolated from headspace or rectal glands); in this framework, dynamic systems like the Y-tubes, allowing an air flow carrying the chemicals to be evaluated for the chemo-ecological role should be preferred, since they avoid the risk of saturating receptors of the tested flies, which is common in still-air arenas. Flight tunnels, also known as wind tunnels, are extremely useful to monitor medium-distance flight responses to mate-derived chemicals [201,270,321]. Their use, for example, allowed to assess the attraction of females to male emissions in *C. capitata* [270] and *A. serpentina* [193], or of both sexes to ME-fed *B. dorsalis* males [70]. Wind tunnels also permitted to prove that pheromone components can exert behavioural effects in *A. ludens* females [181]. These systems can integrate different components for the simultaneous identification of released volatile chemicals and the assessment of their attractiveness, as well as recording environmental parameters and fly activity [201], including the observation of flight patterns in females responding to male-derived volatiles [321]. Lastly, multi-arm olfactometers have been mainly used so far to test fruit odours and with flies released into an area composed of multiple chambers from which airflow-containing odour flows [322].

Moving laboratory results to the field for real-world applications is a timely challenge in tephritid research. Therefore, field assays to evaluate tephritid attraction should be considered after successful laboratory evaluation of a given compound. Field studies can be performed either using live insects/glands extracts as a source of pheromone or traps with different types of dispensers releasing pheromone pure chemicals or mixtures, as described especially for the medfly and the olive fruit fly. In the case of *C. capitata*, a synthetic blend releasing three male pheromone compounds (i.e., ethyl (*E*)-oct-3-enoate, geranyl acetate and (*E,E*)-α-farnesene), in a ratio similar to that observed in natural conditions, was effective in attracting females, as shown by trap catches [225]. Another study found that trimedlure was more effective than pheromone individual components or mixtures in trapping flies [323]. For *B. oleae*, field experiments aimed at determining the attractive effect of the four major pheromone components stressed the importance of finding the ideal combination between attractant formulation and trap type/colour [324]. The use of polyethylene vials as dispensers of either the complete pheromone blend, racemic mixtures of the major components, or individual synthetic chemicals resulted effective in trapping *B. oleae* flies [319,325]. Open field tests were performed either with wild medfly females [225] or released *C. capitata* and *A. suspensa* females [326,327,328,329]. Tests in field cages with potted trees have also been performed, providing valuable information about behavioural responses to live conspecifics or male extracts in seminatural conditions for *A. obliqua*, *A. ludens* and *A. suspensa* [188,244,330].

#### 3.4.2. Reverse Chemical Ecology Approaches

In addition to the above-described techniques to identify tephritid semiochemicals, recent studies are increasingly showing that RCE has great importance in understanding the molecular basis of insect chemical perception and identifying the active volatile semiochemicals [257]. Studies began to be performed to identify genes involved in chemosensory perception in tephritids, including olfactory, ionotrophic and gustatory receptors (ORs, IRs and GRs, respectively), OBPs, odorant degrading enzymes (ODEs), and chemosensory proteins (CSPs) [331]. Insect OBPs are small soluble proteins mostly found in the chemosensillar lymph of sensory organs where they bind molecules of odorants and pheromones (see [332] for a review). Thus, OBPs are considered ideal molecular targets for binding assays to identify chemicals with a potential behaviourally-active role in tephritid biology. The expression of OBPs, followed by their purification and structural analysis, is indeed adopted to perform OBP ligand binding studies; ligands are screened from sets of volatiles emitted from host plants, pheromones, or synthetic attractants used in field applications, and once identified, behavioural responses are evaluated *in vivo* [256].

##### Identification and Functional Analysis of OBP Genes

Putative OBP genes have been initially identified through expressed sequence tag (EST) approaches in *C. capitata* [333], *B. dorsalis* [334,335], *R. suavis* [336]*,* and *R. pomonella* [337] and their transcriptional profile started to be explored. Subsequently, with the advent of next generation sequencing, more OBPs have been discovered through mining of RNA-seq data and whole genome sequencing in several tephritid species, including *B. dorsalis, B. minax* [104,338,339,340,341], *A. fraterculus*, *A. obliqua* [106,342,343], and *C. capitata* [344]. Antennal proteomics profiling has been applied to *B. dorsalis* to identify differentially expressed genes, including OBP genes, in ME-responsive males [345]. Functional studies have been performed to assess their role in odour perception. These include tracing OBP genes expression profiles in different tissues, developmental stages and in response to maturation and mating, RNA interference (RNAi) combined to electrophysiology to assess the involvement of target OBPs in odour detection, followed by behavioural assays [335,341,345,346,347].

##### Purification and 3D-Structural Analysis of Identified/Expressed OBPs

The binding specificity of OBPs expressed in the main chemosensory organs, i.e., antennae and maxillary palps, may help in the identification of pheromone/pheromone components that are still uncharacterised. Thus, OBPs that are (i) abundant in olfactory tissues, or, ideally, specific to these tissues, and (ii) showing sequence similarities to already characterised proteins known to be involved in chemical communication in other insects can be functionally characterised by using ligand-binding assays. OBPs are expressed in bacterial or yeast systems and the recombinant proteins purified with chromatographic steps using different techniques, including anion-exchange or gel filtration chromatography, or affinity chromatography on nickel columns when histidine-tags are added to the OBP sequence (see [348] for a review). Purified proteins can then be used for ligand-binding experiments and to solve their structure through X-ray crystallography or NMR spectroscopy [348].

The first, and, so far, the only available, structural characterisation of a tephritid OBP was recently obtained in the medfly using X-ray crystallography [349]. The structure of CcapOBP22 is characterised by six α-helical elements, a typical feature of insects’ OBPs, interconnected by three disulphide bridges. Differently from other insect OBP structures, CcapOBP22 also carries a 7th α-helix at the C-terminus, which contributes to delimit the ligand-binding pocket. CcapOBP22 was co-crystallised with (*E,E*)-*α*-farnesene as ligand, further supporting the potential role of this protein in semiochemical perception in this species.

##### *In vitro* and *in silico* OBP: Ligand Binding Assays

Several approaches have been adopted to measure the affinity of OBPs to odorants (see [350] for a review). The most common method, which is fast and requires a limited amount of protein, is based on the use of fluorescent reporters, such as 8-anilinonaphthalene-1-sulfonic acid (ANS) and *N*-phenylnaphthalen-1-amine (1-NPN) in competitive binding experiments [351,352,353]. 1-NPN is a lipophilic crystalline solid that strongly binds insect OBPs [354]; when increasing amounts of a tested ligand are added to the OBP/1-NPN system, decreasing 1-NPN fluorescence emission is inferred as 1-NPN displacement since the ligand is assumed to compete for the binding pocket initially occupied by the fluorescent reporter.

This approach has been used in the medfly to evaluate the binding affinity of CcapOBP22 and CcapOBP24 to electrophysiologically-active components of the male pheromone, as well as to the two synthetic attractants trimedlure and ME [223,349]. The finding that also ME, which is a strong attractant for some *Bactrocera* species [170,355,356] but not for medfly, displays binding activity (although moderate) to the above medfly proteins is intriguing. Methyl eugenol is known to induce an electrophysiological response in medfly [302]. In *Z. scutellatus*, ME elicits significant electrophysiological responses too, but it is not behaviourally active [357]. In medfly, ME has been shown to induce poor behavioural responses in binary choice bioassays, while *o*-eugenol was instead strongly attractive [302]. Thus, it appears that the presence of substituents on the aromatic ring can be essential to confer attraction to chemical compounds. It will be interesting to further explore the chemistry of candidate molecules able to bind tephritid OBPs to shed new light on structures that can be exploited as novel attractants. Both OBPs showed the highest binding affinity to (*E,E*)-α-farnesene, which is one of the major components of medfly male pheromone bouquet, and is known to attract females [321], suggesting its role as a natural ligand for these OBPs. The verification of the behavioural responses to the presence of ligands *in vivo* is essential to identify volatile semiochemicals with active roles in fruit fly behaviour.

Ligand-binding assays have also been performed in *B. dorsalis* using 13 chemicals, including pheromone components and attractant molecules, and six proteins with high expression in the antennae (five OBPs and one CSP) [318]. Authors showed that OBPs displayed the highest affinity to the attractants, and, in the case of BdorOBP83a-2, RNAi led to a decrease in neuronal responses to tested molecules, as shown by EAG recordings and behavioural responses.

Computational reverse chemical ecology (CRCE) is another method applied to the discovery of behaviourally active chemicals [358,359]. OBP sequences can be exploited for 3D model prediction, producing 3D structure for docking studies using specific tools. Molecular docking is commonly employed in pharmaceutical research for structure-based drug design [360,361]. It implies the use of programs based on different algorithms applied to model the interaction between a small molecule and a protein at the atomic level. This allows the exploration of the behaviour of small molecules in the binding pocket of target proteins [362]. CRCE has been implemented in *B. dorsalis* to screen 25 chemicals for their binding potential to a general OBP (GOBP) showing that this approach may be extremely useful to quickly predict behaviourally-active semiochemicals, for example selecting chemicals belonging to specific classes [358]. The described approach is particularly beneficial especially in tephritids given the wide absence of direct crystallographic data for OBP binding modes.

##### Identification and Functional Analysis of OR Genes

Although OBPs are excellent study targets to either understanding the molecular and biochemical mechanisms of odour perception in insects, and to explore the development of pest control agents, they have broad binding specificity, are also distributed in non-olfactory tissues and have different functions [363]. Conversely, ORs are transmembrane proteins showing high specificity and sensitivity. Thus, genes encoding for chemosensory receptors are also becoming to be identified and characterised in tephritids, such as *B. dorsalis* [338,364,365,366]*, B. minax* [341]*, B. oleae* [367]*, B. latifrons* and *Z. cucurbitae* [368]*,* and *C. capitata* [344]. Olfactory receptors have been described as heteromeric ligand-gated ion channels consisting of a specific OR and the highly-conserved co-receptor Orco [369]. Olfactory receptors are transmembrane proteins for which no 3D structure is available yet, and they are a more difficult target than OBPs to be expressed and purified in heterologous systems. So far, the only three-dimensional structure currently available, obtained using a cryo-electron microscopy-based approach, is for a tetramer of Orco, described in the parasitic fig wasp *Apocrypta bakeri* (Joseph) [370]. Thus, only limited data on the functional activity of tephritid ORs are available. In a recent study, ten *B. dorsalis* ORs were co-expressed with their essential co-receptor BdorORCO in *Xenopus laevis* Daudin (Anura: Pipidae) oocytes. Two-electrode voltage clamp was then used to record currents from injected oocytes when ligands (i.e., 1-octen-3-ol, geranyl acetate, farnesenes, and linalyl acetate) were diluted in the assay buffer [365]. Some of the identified ORs have been shown to respond to plant volatiles [365] or ME [371]. Further research efforts oriented to clarify the structure of tephritid ORs are essential to understand the molecular recognition mechanisms they are involved in, as well as their interactions with OBPs, and thus their functional roles. Insect ORs display a different topology from those of other animal G protein-coupled receptors (GPCRs) [372], with a C-terminal faced to the extracellular section and the N-terminal to the intracellular section. This feature makes insect ORs ideal targets to be explored for the development of insect-specific pest control strategies. These may include the inhibition of either Orco or the ORx/Orco complex by antagonists able to, for example, disrupt mating behaviour through the manipulation of pheromone receptivity. Interestingly, in *B. oleae*, transient knockdown via RNAi gene silencing in adult individuals showed that knockdown of Orco expression reduces the mating ability in both sexes and completely inhibits oviposition [367].

Tephritid CSPs have been identified [336,338,373], but their variable pattern of tissue distribution, the different potential functions, the still unproven binding ability [318] and unavailability of structural information [363] is locating them in a less attractive field of investigation.

## 4. Host-Marking Pheromones

### 4.1. Chemical Identity, Production and Analytical Approaches to Their Characterisation

In tephritids, host-marking behaviour was first described in *R. pomonella* [374]. Later, Hafliger speculated that the biological role of this behaviour was to equally distribute the offspring in available host fruits [375]. Following these earlier observations, Prokopy and Cirio were the first that experimentally described in 1972 the HMP deposition in *R. pomonella* [112] and *R. completa* (Cresson) [135]. Since then, the host marking behaviour has been reported in 25 tephritid species, particularly frugivorous species, belonging to the *Anastrepha*, *Ceratitis* and *Rhagoletis* genera [114] (Table 3; Table S3).

However, it is not a general feature of the family; it seems to be common in *Rhagoletis* spp., sporadic in *Anastrepha* and *Ceratitis* spp. and rather absent in others (e.g., *Bactrocera* spp.) [29,35,129,390,391].

HMPs are low-volatility and highly polar molecules [392]. They are also soluble in water and methanol [30,131,382,393,394]. HMPs can persist on the surfaces either when they are directly deposited by the fruit flies or as extracts [108]. For instance, the HMP half-life has been estimated to 10.7 days with persisting activity for three weeks in *R. pomonella* [383], 9 days in *R. fausta* [387]*,* 12 days in *R. cerasi* [386], 6 days in *A. suspensa* [376]*,* 6 days in *C. capitata* [111] and 4 days in *R. indifferens* [392].

To the extent of our knowledge, HMPs are produced and stored in the posterior half of the midgut and, thus, the faecal matter contains a huge quantity of these pheromones, suggesting the existence of two main routes for HMP deposition: through ovipositor dragging after egg-lying and through defaecation [132,395,396]. In *R. pomonella*, HMP accumulates in the midgut, Malpighian tubules, hindgut and faeces of mature females [395].

HMPs have been isolated from faecal matter extracts using approaches based on liquid chromatography (LC) and MS, in all four species (Table 3), namely HPLC-FAB-MS (Fast Atom Bombardment Mass Spectrometry) for *A. ludens* [382] and *R. cerasi* [30], and LC-quadrupole time-of-flight-mass spectrometry (LC-QTOF-MS) in the case of *Ceratitis* species [390,397]. Therefore, all the HMPs that have been chemically identified were isolated from the aqueous or methanol extract of adult female faecal matter. To date, it remains to be determined whether HMPs are produced by specific glands [395].

HMP chemical identity has been so far determined in a few tephritid species (Table 3). The first chemical characterisation of an HMP was achieved in *R. cerasi*. The pheromone was a complex molecule, i.e., N-[15(*β*-glucopyranosyl)-oxy-8-hydroxypalmitoyl]-taurine, with four stereoisomers [30], showing two chiral centres at the C-8 and C-15 positions. After the synthesis of the four different stereoisomers [398], it has been demonstrated that a racemic mixture of two isomers (8*R*, 15*S* and 8*S*, 15*R* isomers) is able to deter oviposition [399].

Later, the HMP [2-(2,14-dimethylpentadecanoylamino)pentanedioic acid (or *N*-[2,14-dimethyl-1-oxopentadecyl)glutamic acid)] of *A. ludens* has been chemically characterised and synthesised [382,400]. It presented a relatively simple structure containing an isopalmitic fatty acid chain substituted by methyl at the C-2 position and coupled to glutamic acid (GA) as a single diastereomer [382,400]. The HMP of *A. ludens* exhibited not only intraspecific but also interspecific oviposition deterring activity to *A. obliqua* and *A. serpentina* [132]. Of note, the HMPs of *R. cerasi* and that of *A. ludens* display similarities in structure (i.e., both contain a long fatty acid residue attached to an amino acid).

Recently, the HMP of *C. cosyra* and *C. rosa* have been isolated and both chemical structures have been determined [33,34]. The *C. cosyra* and *C. rosa* HMP identified are the tripeptide glutathione (GSH) (consisting of glycine, cysteine, and GA [33]), and GA [34]*,* respectively. Interestingly, GSH and GA levels were 5–10 and 10–20 times higher in the faecal matter than in the ovipositor or haemolymph extracts of the respective females. These results suggest that the HMPs may be transferred from the gut into the ovipositor through the haemolymph and the excess amount may be expelled with the faecal matter. GSH was shown to express pheromone and allomone action respectively, reducing the oviposition in individuals of the same species and in those of different species, such as *C. rosa*, *C. fasciventris*, *C. capitata*, *Z. cucurbitae*. Interestingly, the GSH acts as kairomone inducing arrestment behaviour in the egg parasitoid *Fopius arisanus* (Sonan) (Hymenoptera: Braconidae). On the other hand, GA perception resulted in oviposition reduction in *C. rosa* and *C. fasciventris*, but not in *C. cosyra*. It is noteworthy that the HMPs of the two *Ceratitis* species are highly distinct from the HMPs identified in the other fruit flies. However, they appear to be more closely related to the HMP of the Mexican fruit fly, which contains GA, than of *R. cerasi* that is a fatty acid glucoside. Taken together, these findings may indicate that closely related species may utilise a similar pathway for HMP synthesis.

Host marking through HMP deposition is noticeably absent in the *Bactrocera* genus, even if contrasting results have been reported [35,139]. To prevent other females from ovipositing on the same fruit, *B. oleae* females do not depose an HMP but use their labella to spread olive juice leaking from the oviposition wound, with the main compounds responsible for this repulsion being (*E*)-hex-2-enal and oleuropein derivatives, such as the hydroxytyrosol [143,401].

The molecular machinery underlying the perception of these substances by other females remains to be determined. However, early studies in *R. pomonella* suggested that D-hairs on specific segments of the ventral tarsal surface and short marginal hairs on the labellum carry the receptors for HMP detection [395].

### 4.2. Behavioural Assays

Behavioural studies to assess the ecological role of a potential HMP rely on dual choice oviposition assays conducted under a completely randomised design [111], where tephritid females can choose to oviposit on a fruit marked by conspecifics over a control fruit [33,402].

## 5. Cuticular Hydrocarbons

Cuticular hydrocarbons act as pheromones in a variety of orders, including Diptera [25,403,404,405]. Their behavioural function in flies was first described in the housefly, *Musca domestica* L. (Diptera: Muscidae), where (*Z*)-tricos-9-ene was identified as the main compound on the female cuticle acting as a sex pheromone for males [403,406,407,408]. Extensive evidence of the importance of hydrocarbons (7-monoenes) as short-range signals and contact pheromones comes from *Drosophila* spp. [23]. These semiochemicals are perceived by antennae and maxillary palps and/or by contact with the taste organs that are mostly located on the tarsi and proboscis [409,410,411]. *Drosophila* spp. show relatively stable CH profiles, although their production can vary as the flies age and even after reproductive maturation [412,413]. In tephritids, sex- and species-specificity of CHs have been described in species of the genera *Anastrepha*, *Bactrocera*, *Ceratitis* and *Zeugodacus* (Table 4; Table S4).

Characteristic CHs profiles have been successfully applied for the chemotaxonomic clarification of fruit fly species complexes of *A. fraterculus*, *B. dorsalis* and the so-called African *Ceratitis* FAR complex [90,274,414,416,417,418,420,421]. Nevertheless, studies focused on the elucidation of CHs behavioural function in fruit fly mating system are still missing, except for those performed on *B. oleae* [80,85,288].

### 5.1. CHs in Tephritid Species and Their Described Roles

Earlier comparative studies on adults of *A. ludens*, *A. suspensa*, *C. capitata*, *C. rosa*, *Z. cucurbitae* and *B. dorsalis* failed to identify substantial sex-specific differences in CH profiles [414,420]. It seems that the only difference detected is the much higher amount of *n*-alkanes in males compared to females in both *B. dorsalis* and *Z. cucurbitae* [420]. However, further research reported sex- and age-dependent differences in CH production for a laboratory population of *A. fraterculus* (Brazilian-1 morphotype). Sexually mature males have specific unsaturated hydrocarbons (7-monoenes) on their cuticles that lack in females [90,289]. In follow-up studies, sexual dimorphism has been evaluated in the Brazilian-1 (Argentina), Brazilian-3, Andean, Peruvian and Mexican morphotypes of the *A. fraterculus* species complex [92,93]. The Brazilian-1 morphotype expresses the highest sexual dimorphism (29.46%), followed by the Mexican (15.42%) and Peruvian (13.79%) ones [93]. Males and females from the five abovementioned morphotypes diverge in alkene and alkadiene content. In *A. ludens,* the age-dependent CHs production in males originated from a standard mass-reared colony, a genetic-sexing strain, a hybrid strain and a wild population has been recently described [415]. Wild males of *A. ludens* differ from the mass-reared strains in the amount of nonacosane, while genetic sexing strain expressed higher values of 2-methylhexacosane. It has been suggested that the observed differences in CHs profiles may be due to environmental pressures [415], but additional research efforts are still needed to clarify these issue.

Further research also focused on the CH composition of males and females of the African fruit fly cryptic species FAR complex, demonstrating that sex-specific differences in the CH composition do exist [214,274]. The CH sex-specificity was proved by multivariate statistical analyses of the GC×GC-MS data of 59 CHs identified in the epicuticular extracts of *C. capitata*, *C. fasciventris*, and *C. rosa*. In contrast, the cuticular profiles of *C. anonae* display no sex-specificity [274].

In species of the *B. dorsalis* complex, abundant complex mixtures of sex-specific oxygenated lipids (i.e., three fatty acids and 22 fatty acid esters) with so far unknown biological function were identified in epicuticle extracts from females [421]. Such sex-specificity may be driven by sexual selection if the chemical composition of the cuticle is used as a pheromone signal in mate choice. Although early studies suggested that *B. dorsalis* males are able to recognise females at short distance and that physical contact may play key roles in courtship and *copula* [424], only limited functional information are currently available. These include the data describing the strong attractiveness to males exerted by the female-specific CH 4-allyl-2,6-dimethoxyphenol (4-DMP), which has been regarded as close-range sex pheromone [425]. This compound elicits electrophysiological responses in the mid legs of *B. dorsalis* males [426]. Moreover, after stimulation with 4-DMP, five OBP genes are found upregulated in males, with one of them, BdorOBP2, being a promising candidate for the binding and transport of 4-DMP [426], in addition to its proposed role in the perception of ME [345]. Recently, the cuticular components of *B. tryoni* have been described [423]. The spiroacetals and esters were found to be female-specific, while amides were presented in both sexes. Nevertheless, the role of these and other CHs in short-range chemical communication of *Bactrocera* spp. and other fruit flies needs to be further elucidated through expansion of the molecular machinery underlying their production and perception and with proper behavioural assays.

### 5.2. Analytical Approaches to Trace CH Profiles

Preparation of samples for gas chromatographic analysis of tephritid CHs is usually made by solvent extraction of whole insect bodies with solvents such as pentane, hexane, dichlormethane, and chloroform [90,427]. The most widely used method for recovering tephritid CHs is hexane washing [274,421,428]. This process may contaminate the cuticular sample with other materials, such as those from the exocrine glands. To collect only the CH fraction of the extract, the hexane solution is placed on a short chromatographic column and the CHs eluted with a small volume of hexane. For pre-cleaning of the CHs, thin layer chromatography (TLC) has proven useful in identifying novel lipid pheromones. TLC plates consist of glass or aluminium coated with an adsorbent layer of silica gel. Using TLC, components of the chemical extract can be separated into discrete fractions according to hydrophobicity [429,430]. After the hydrocarbon fraction has been collected, the solution must be concentrated to a suitable volume before the analysis. For the determination of the final concentration of the sample, the number of insects extracted must also be known. Another reported method involves continuous extraction of the insect in a refluxing solvent [431]. This procedure also requires column chromatography and re-concentration of solutes. Solvent extraction procedure involves several steps, in which more volatile compounds may be lost during the process, and moreover the use of high-purity solvents is required. Besides, the extraction and sample preparation account for most of the analysis time [428].

The solvent-free SPME technique has been used for the analysis of cuticular components of *Trupanea vicina* (Wulp), an Asteraceae-feeding tephritid [432]. SPME polydimethylsiloxane coated fibre (PDMS) was used to wipe samples from various body parts of male *T. vicina* and the subsequent GC-MS analyses showed that 1-nonanol, the male-specific compound, was concentrated on the abdomens of males exhibiting pleural distension [432]. In *A. ludens*, a PDMS fibre was used to rub down the male wing CHs, which were subsequently analysed by GC-MS [415]. Recently, the direct immersion-SPME (DI-SPME) coupled with GC-MS analyses was employed for characterisation of *C. capitata* semiochemicals in three different mating stages. This study demonstrated that medfly compound compositions were not significantly different before and during mating. However, new chemical compounds were generated after mating, such as (*Z*)-tricos-9-ene and hentriacontane among ten other components [260]. Female medflies seem to discriminate mated from virgin conspecifics and express higher rates of aggressive behaviour against the later [433]. Considering the recent findings, it is plausible to argue that changes in CHs of mated females drive the differential aggressive interactions between virgin and mated female medflies.

The method of choice for the analysis of the CH profile in tephritids has been GC-MS or GC×GC-MS using two different ionization techniques: electron (EI) and chemical ionization (CI) [93,289,427,434,435]. EI-MS is the primary tool for assessing the location of methyl branch points in long-chain alkanes, but it is often difficult to identify the molecular ion. The EI mass spectra interpretation allows complete identification of a compound, but often microscale reactions or derivatizations are necessary to provide additional structural information [436]. A mass spectrum also provides a ‘finger print’ of a compound, which can be compared with the libraries and mass spectra registries such as the NIST library, the Wiley/NBS registry of mass spectral data, and the published retention indices [437,438]. Mass spectra of an CI-MS yields the (M-1)^+^ ion as an intense peak (sometimes as the only peak), but when the peak consists of mixture of isomeric methyl or dimethyl alkanes, placement of methyl group position becomes more difficult [435]. Another problematic aspect is the determination of the double bound position using EI-MS, because of the lack of cleavage between carbon-carbon double bonds or extensive and facile hydrogen rearrangement along the chain after molecular ion fragmentation [439]. Nevertheless, several works documented the CI-MS/MS with acetonitrile ionization gas to be a suitable method for double bound position determination [90,434,440].

There have also been studies using MALDI-TOFMS and ultraviolet laser desorption ionization orthogonal time-of-flight mass spectrometry (UV-LDI o-TOFMS) for tephritid CHs identification [90,429,441]. Cvačka and colleagues [429] applied MALDI-MS for the identification of insect CHs, using lithium 2,5-dihydroxybenzoate as a matrix [442]. This work demonstrates that MALDI-TOFMS is a convenient analytical method for the identification of high molecular weight hydrocarbons from insect cuticles, including saturated hydrocarbons and highly unsaturated and/or cyclic compounds. In *A. fraterculus,* application of MALDI-TOFMS method allowed for characterisation of high molecular weight saturated and unsaturated hydrocarbons, up to C37 in length [90]. Mass spectrometric imaging (MSI) of male and female *A. fraterculus* was performed using a previously developed protocol [443]. The preliminary MALDI-MSI experiments indicate differences in the CH distribution on the wings of males and females [289]. Nevertheless, additional detailed analyses using MALDI-MSI techniques are necessary for further conclusions concerning the CHs of *A. fraterculus*. The imaging data will show if some of the CHs have unique locations on *A. fraterculus* body surface and can also indicate if the compounds direct male/female sexual contacts.

### 5.3. Behavioural Assays

Behavioural tools currently used for the evaluation of CHs do not substantially differ from the set up described above for volatile pheromones. Indeed, observation chambers, as well as two- or multiple-choice systems (i.e., Y-tube/T-maze and multiple-arm olfactometers, respectively) are widely used to evaluate behavioural responses triggered by CHs with potential pheromone activity [70,75]. Further validation of the observed behavioural response can be documented in flight tunnel as well as in field and semi-field assays [171,319,444].

As a final remark, semiochemical candidates, such as potential volatile pheromones and CHs, could be evaluated for their biological functions relying on the mixed society approach [445]. Indeed, mixed societies composed of living insects and small-sized robots mimicking their conspecifics can represent a valid approach to shed light on factors guiding insect behaviour, including mating approaches. This ethorobotics-based approach has been validated on several insect species, such as cockroaches, beetles and blowflies [446,447,448]. However, no studies have been conducted relying on ethorobotics in tephritid research. In our opinion, this represents a challenge for future studies.

## 6. Tephritid Sexual Chemoecology: Real-World Applications and Challenges

Semiochemical-based interactions have been extensively studied in tephritid fruit flies and several aspects of the generated knowledge had already been exploited for practical purposes including (a) trapping and population monitoring, (b) direct population control approaches (lure and kill methodologies, and push and pull strategies), and (c) support and improvement of other methods, such as the SIT. More recent progress in the characterisation of (a) the olfactory molecular machinery, including OBPs and ORs, and (b) CHs may open new venues in developing inspiring approaches for artificial olfaction and hence generation of novel long- and medium-range attractants. These tools can be used to address issues regarding species complexes that may be of vast importance for regulatory and control aspects. Nonetheless, a deeper knowledge of semiochemical-based communication is essential to further understand how tephritid species adapt to complex ecosystems, also with respect to the invasive potential of several pests belonging to this family, further advancing applied research from several perspectives.

### 6.1. Population Monitoring and Early Detection of Tephritid Outbreaks

Given the influence semiochemicals play on insect behaviour, a better understanding of their identity, specificity and biological role(s) may benefit tephritid research by developing novel specific and environmentally-friendly attractants. Effective attractants are essential for adult trapping, which is a key tool used to (a) monitor density and seasonal patterns of established tephritid pest populations, (b) detect new infestations of exotic species, (c) delimit the detected populations, and (d) confirm the results of eradication campaigns. Early detection of small populations is particularly important to delimit the outbreak and thus implement control and eradication measures while the pest population is still present at low densities [449,450].

The list of odour attractants for tephritid fruit flies is quite long including food-based (protein-based, ammonia releasing compounds) and mating-related chemicals. The later can either be purely synthetic or derived from plants. The history of their discovery and development goes beyond the purposes of this review, and their identity, efficacy, and the related practical aspects of their use in monitoring and detection are widely reviewed in [171]. Attractants that are related to the mating behaviour or physiology of fruit flies have been more thoroughly studied in the case of *Bactrocera* and *Ceratitis* genera and they exclusively concern male lures. For example, both the plant derived *α*-copaene and the synthetic trimedlure and Ceralure are highly attractive to male but not female medflies [171,451,452]. Interestingly, both plant-derived and synthetic attractants are related to male reproductive success and may enhance lekking behaviour and mating competitiveness [453,454,455,456,457,458]. It should be stressed here that trimedlure is rather the most commonly used attractant for medfly regular population monitoring, control programs and in detection and eradication campaigns [459,460].

Some *Bactrocera* species respond to Cue Lure (CL)/RK, others to ME, the most powerful attractant so far identified, others appear to be non-responsive to both. Eugenol analogues (isoeugenol, methyl-isoeugenol and dihydroeugenol) are also proving successful in attracting *Bactrocera* species [461,462]. Similar to species of the genus *Ceratitis,* the above compounds attract only males of the *Bactrocera* species and again are related to their mating success [463,464,465,466,467,468]. Methyl eugenol is considered one of the most powerful attractants for male fruit flies and it is extensively used in detection, population monitoring, delimitation and eradication campaigns worldwide [170,469,470].

Conversely, no male lures are currently available for *Anastrepha* and *Rhagoletis* spp. [171]. Exploring the potential of tephritid pheromones to develop novel specific attractants is thus important, not only for *Anastrepha* and *Rhagoletis*, but also for non-responsive species belonging to the *Bactrocera* and *Ceratitis* genera. Moreover, ME has been suggested to be carcinogenic [471] and alternatives are required that also do not exert effects on non-target species. Finally, the available lures are generally effective in attracting one sex, and do not have a species-specific action.

Although food-based attractants [472] are still dominant in population monitoring of the olive fruit fly, the use of the female pheromone can provide additional information on both the population density of wild populations and the age structure of wild populations. More recently, Sarles and colleagues identified two lactones released exclusively by males of *R. completa* and used lactone-baited traps in walnut orchards [473]. These traps have been found particularly effective for *R. completa* monitoring and allowed its earlier detection in the season, supporting the idea that the analysis of pheromone components may be particularly promising for trapping tephritids in the field.

### 6.2. Eradication and Suppression of Fruit Fly Populations Employing Semiochemicals

Male lures such as trimedlure and especially methyl-eugenol (ME), besides being employed for population monitoring, have been used for more direct control purposes. Male annihilation technique (MAT) (i.e., elimination of males, mating and oviposition of fertile eggs, based on strong male-specific lures that are deployed in a mass trapping or lure and kill approach) is considered as a very successful option to eliminate low populations of *B. dorsalis* and a tool that may drive invaded population to extirpation or even eradication. For example, male annihilation against *B. dorsalis* has been used as a main tool in attempts to eradicate incursions or isolated established populations in California, Hawaii and Florida [474,475,476,477,478], South Africa [479,480], the Marianas Islands in Micronesia [481], the Okinawa Islands in Japan [482], and Mauritius [483]. Eradication efforts using the male attractant CL against other *Bactrocera* species such as *B. frauenfeldi* were not successful [484]. Male attraction to CL remained consistent until advanced age in *B. tryoni*, although it sharply declined after 12 weeks of age, with potential implications for pest management [485]. Despite its broad use as a population monitoring tool, trimedlure is not considered as an eradication tool against medfly.

Male lures and the MAT have been considered in suppression programs often in combination with other methods and preferably in the frame of an Area Wide application strategy. As it was demonstrated in Hawaii, combination of MAT with field sanitation, protein bait, sterile male releases and biological control resulted in satisfactory reduction of the fruit fly population [486]. Methyl eugenol and CL for *Bactrocera* species, as well as trimedlure for the medfly, have been considered. Interestingly, the use of ME for the suppression of *B. dorsalis* in Southern Ethiopia proved to be successful [487].

The use of the female pheromone alone or in combination with other baits has been evaluated for the control of the olive fruit fly [488,489]. These efforts include a male annihilation component but also tools against females.

Apparently, classical mating disruption approaches involving saturation of target area with species-specific pheromones are not effective against fruit flies, and the MAT is prevailing. Indeed, mating disruption approaches against *B. oleae* in Spain and Greece led to inconclusive results [490,491]. However, a more recent study, in which authors observed a decrease in fly catches in the presence of high pheromone (i.e., 1,7-dioxaspiro[5.5]undecane) concentrations, supports the applicability of a mating disruption approach against this species [492]. For the medfly, for both trimedlure and the iodinated trimedlure analogue Ceralure, no mating disruption effects have been so far described [492]. Such an absence of disruption effect in this species was explained by the lack of saturation in response to higher trimedlure concentrations [492].

Alternative strategies aimed at interfering with mating deserve to be explored, including the potential applications of CHs as disruptors. CHs are known to play a role in the mating behaviour of different *Drosophila* species [493,494,495]. In *D. suzukii*, alteration of C23 alkanes ratios results in disrupting mate recognition and, as a consequence, courtship and mating behaviour [496]. The identification of OBP candidates potentially able to transport the *B. dorsalis* female-biased CH 4-DMP [425,426] is a promising step in the clarification of the functional role of CHs in the mating behaviour of fruit flies.

Moreover, in order to improve the efficacy of any semiochemical-based approach for fruit fly eradication and suppression, additional studies should be devoted to better understand the role of abiotic factors on trapping. Indeed, although temperature, humidity, rainfall and other exogenous abiotic factors do affect the temporal and spatial activity of fruit flies, data about the role of such factors in tephritid captures are still limited and mostly available for liquid protein-baited traps (see [269] for an overview). In the case of semiochemicals, it is known that medfly attractiveness to trimedlure is related to the release rate of this compound, which is, in turn, dependent on temperature [497,498]. Recently, Cameron and colleagues examined the vapour pressures and thermodynamic properties of seven attractants (i.e., RK, CL, raspberry ketone trifluoroacetate-RKTA, ME, methyl isoeugenol, dihydroeugenol, and zingerone) currently used for trapping *Bactrocera*, *Dacus* and *Zeugodacus* species [499]. The authors provide valuable data regarding the volatility of these attractants. In particular, they found that (*Z*)-methyl isoeugenol is the most volatile of the ME-type compounds, while RKTA is the most volatile among the RK-type compounds. Interestingly though, the field life of RKTA is not long due to its susceptibility to humidity [500]. Expanding our understanding of the features of these chemicals as well as of the identity/impact of multiple abiotic factors that may affect their activity is essential to determine the design of the eradication and suppression programs, as well as the location and density of the traps to be used.

### 6.3. Push and Pull Approaches Based on Repellent Semiochemicals

HMPs have been regarded as attractive tools for tephritid pest control since the ‘70s. In 1976, Katsoyannos and Boller proposed to use HMPs to prevent fruit fly oviposition into the fruit. They performed the first field experiment spraying raw HMP extract obtained from the faecal matter of *R. cerasi*. In this way, they achieved over 90% reduction of *R. cerasi* infestation in cherries orchards [501,502]. Later, once the chemical structure and the synthesis of *R. cerasi* HMP have been obtained [29], Aluja and Boller [31] tested the synthetic *R. cerasi* HMP in the field and, interestingly, this was the first application of a “push-pull strategy” in fruit flies. A push-pull strategy exploits a combination of behaviour-modifying stimuli to manipulate the distribution and abundance of the insect targets. Pests are repelled from their resource (push) by using stimuli that mask the host or that acts as repellents. Simultaneously, they are driven away from the resource (pull), by using highly attractive stimuli such as traps, facilitating their elimination. A reduction of the infestation of about 90% in cherry plants was achieved by treating one half of tree canopies with a synthetic HMP. The repelled females were then trapped with visual traps placed on the other half of the canopy [31,503]. The efficacy of the synthetic HMP was further supported by another field trial in which the infestation by cherry fruit flies was eliminated [504].

Similarly, field tests were performed using raw pheromone extract from the medfly’s faecal matter achieving 84% decrease in infestation in sprayed coffee plants [390]. Exploiting the cross-recognition observed in the *Anastrepha* genus, Aluja and co-workers tested three potential oviposition deterrents for *A. obliqua* in tropical plum and mango orchards. In their experiments, they used *A. ludens* faecal extracts and two fully synthetic simplified analogues of the naturally occurring pheromone, namely desmethyl *A. ludens* HMP (DM-HMP) and anastrephamide. They obtained a significant reduction in fruit damage rates with all substances tested, and interestingly, the simplified analogues displayed comparable levels of efficacy to the natural HMP [32].

The good efficacy of the synthetic HMP analogues found in both *R. cerasi* and *A. obliqua* field tests is promising in view of their potential use as pest control strategy. However, there is evidence that, after prolonged exposure to the HMP, flies can lay eggs in the treated fruits. This behaviour could be associated with the sensorial adaptation by the insect [123,503].

Although the use of HMP in the management of fruit flies was initially suggested as a push-pull system [32,112,393,400,505], the push-pull strategy is not suitable for the species with high population growth rates [506]. A recent work used anastrephamide in combination with a protein bait to reduce grapefruit infestation by *A. ludens* [507]. The authors found that anastrephamide can push flies out of the treated tree, but the push-pull system requires a more effective attractant.

Lastly, an alternative use of HMP has been proposed, which implies HMP application in commercial crops in which the fruit fly populations are not resident. This option allows achieving pest suppression because of the small population and lower risk for the occurrence of adaptation [114].

### 6.4. Implications for Biological Control

The recent findings that fruit fly parasitoids such as *F. arisanus* responded to HMP of fruit fly species [114,508] may pave the way for the development of methods and approaches to enhance the biological control efforts against fruit flies. Intensifying the research towards understanding the intraspecific interactions among parasitoids and fruit fly semiochemicals, in particular, oviposition-induced volatiles, is required to further increase parasitoid ability to localise and parasitise tephritid pests in the field [509]. In particular, expanding our understanding of the identity of HMPs will facilitate approaches based on the manipulation of parasitoid behaviour to benefit fruit fly control. Indeed, the incorporation of the identified HMP chemicals in the mass rearing process of the target parasitoid has the potential to facilitate the associate learning process that allows the parasitoid to distinguish HMPs from plant-emitted volatiles [114,510]. Moreover, treatments of fields and orchards with synthetic HMPs may not only deter fruit fly oviposition, but also attract parasitoids [511,512]. Among the factors mediating semiochemicals’ production and perception by tephritid and/or parasitoids, the insect microbiota likely plays a key role. Interestingly, a recent study showed that the production of *β*-caryophyllene emitted by host plants and mediating oviposition avoidance in egg-infested fruit by *B. dorsalis* is induced by egg-surface bacteria such as *Providencia* sp. and *Klebsiella* sp. [513]. Achieving a deeper understanding of the interplay between tephritid, their microbiota and host plants will shed new light on the multifaceted field of trans-kingdom communication [514] and will provide novel targets to be exploited for pest management.

### 6.5. Semiochemical-Based Tools to Enhance the Sterile Insect Technique

The identification and functional characterisation of tephritid semiochemicals may be beneficial for SIT applications and can also favour the integration of SIT and MAT to maximise the efficacy of pest management campaigns.

In SIT programs, sterilised males are released in the field to mate with wild females and induce sterility and hence no viable egg production [515]. Typically, sterile males are release as immature adults, which have to survive until sexual maturation, localise females and achieve copulation for SIT programs to be successful [516]. Thus, survival, dispersal capacity and mating competitiveness, for which male signalling is essential [329,517], are key factors that have to be fulfilled. Mating-enhancing semiochemicals have been widely described in several tephritid species and include both plant-derived compounds and synthetic chemicals, such as ME, Cue-Lure, RK, ginger root oil (GRO), citrus and guava fruit volatiles, and manuka oil (see [518,519] for reviews, and [458]).

SIT campaigns are more effective when pest population density is reduced before the release of sterile males. MAT applications, as well as inundative releases of biological control agents, have the potential to reduce the density of feral population size and hence to precede the implementation of the SIT. In *Bactrocera* species, the pre-release exposure of males to plant-derived semiochemicals and synthetic lures has been shown to reduce their subsequent response to attractants used in MAT [35,520]. Recently, the use of a diet containing RK fed to immature sterile *B. tryoni* prior to release resulted in increased subsequent survival and reduced response to MAT [521]. These results are particularly promising for the implementation of SIT-MAT simultaneous used in the field, to both increase control effectiveness and reduce operational costs.

### 6.6. Artificial Olfaction and Pheromone-Based Nanosensors

The ability of insect to detect olfactory stimuli at low concentrations over long distances [522] stimulated researchers to exploit these phenomena to develop biosensors based on insect behaviour [523], using isolated antennae [524] to detect explosives [525,526], food toxins [527], and for disease control and diagnosis [528,529]. Biosensors require a biological and artificial component, able to make the signals readable. OBPs can be expressed and purified easily and are stable to perturbations in temperature, pH and proteases [530]. Thus, they are considered ideal candidates to be exploited in biosensors development and were used to engineer systems able to detect floral odorants, alcohols and explosives in *Drosophila* and *Apis mellifera* L. (Hymenoptera: Apidae) [531,532,533,534]. More recently, also a member of the Tephritidae family was target of this type of research: an OBP from *B. dorsalis*, BdorOBP2, was expressed, purified and immobilised on an interdigitated electrode and it was shown to work as an efficient biosensor for chemicals emitted by host plants (e.g., isoamyl acetate, *β*-ionone, benzaldehyde) [535]. As previously described, given their higher specificity and sensitivity ORs are ideal candidates to be explored for the development of biosensors. Research in the field of OR-based bioelectronic sensors is indeed recently emerging as a simpler strategy with respect to the use of the mammalian ORs to detect environmentally significant volatile organic compounds (VOCs), as shown in mosquitoes (Diptera: Culicidae) and *Drosophila* [536,537,538]. Although interesting, this type of application is still far from being used in the field for tephritid management, also because only a few insect ORs have been deorphanised. Conversely, the exploitation of pheromone components for the development of innovative strategies to monitor early infestations is emerging as a powerful alternative to currently adopted strategies. Indeed, by targeting the major olive fruit fly volatile pheromone component, 1,7-dioxaspiro[5.5]undecane [319], Moitra and colleagues developed a *β*-cyclodextrinylated nanosensor specific to the female volatile pheromone of *B. oleae* [539]. This device is currently being tested in open field conditions and may be important not only for the control of *B. oleae* but also for the development of similar sensitive microelectromechanical system (MEMS) devices for other tephritid pests.

### 6.7. Cuticular Hydrocarbons as a Tool for Chemical Taxonomy

Cuticular lipid profiles, which are species-specific both in solitary and social insects, serve as fingerprints, making it possible to discriminate species taxonomically or to recognise sibling species [443]. The first successful use of CHs for the taxonomic discrimination of tephritid fruit fly species was reported 20 years ago in five articles on the hydrocarbon profile identification of adult Malaysian *B. dorsalis* complex flies [420] and larvae of *Anastrepha* (*A. acris, A.* *fraterculus*, *A.* *suspensa, A. ludens*, *A. obliqua*), *Ceratitis* (*C. capitata, C. rosa*), *Bactrocera* (*B. dorsalis*), and *Z. cucurbitae * [414]. Recently, Vaníčková and co-workers reported CH profiling is an efficient tool for the resolution of entities in the African fruit fly cryptic species FAR complex [274,289,414,416,417,418]. In these studies, twelve potential chemotaxonomic markers were identified for the distinction of adult male and female flies of *C. fasciventris*, *C. rosa, C. anonae* and *C. capitata.* Some of the geographically distinct subspecies hidden in the *A. fraterculus* complex can also be identified using their specific CH profiles. For example, Peruvian and Brazilian-1 morphotypes have unique CH profiles, suggesting CHs could be used to distinguish between these two subspecies [93]. In the *B. dorsalis* complex, clear segregation of complex cuticle profiles of both *B*. *carambolae* sexes from *B*. *dorsalis* was documented, supporting both taxonomic synonymisation of *B*. *invadens*, *B*. *papayae*, and *B*. *philippinensis* with *B*. *dorsalis*, as well as the exclusion of *B*. *carambolae* from *B*. *dorsalis* [421].

## 7. Conclusions and Challenges

Research in the last decades amazingly expanded our knowledge in the field of tephritid fruit fly semiochemical-based communication at the genomic, molecular, physiological and behavioural level, as outlined by the huge amount of available literature. On the other side, it is clear that semiochemical-based communication has not been characterised to an equal extent in all relevant tephritid pests. Moreover, most studies have been focused on volatile pheromones, with far less information available for CHs and HMPs. Thus, a major research need is to expand our knowledge to achieve an exhaustive understanding of all the semiochemical-based communication modalities in the target species. In addition, many specific questions remain to be answered. In the case of volatile pheromones, the application of different techniques and conditions for sampling, as well as the chosen source (i.e., rectal gland content or headspace), often provided different results in the detection and quantification of volatiles, making comparisons among identified sets of chemicals challenging. This urges for the parallel adoption of more than one method to ensure a comprehensive analysis of volatile pheromones. Moreover, future research on volatile pheromones will be essential to clarify (i) the identity of the molecular/genomic machinery underlying rectal (and salivary) gland physiology leading to volatile pheromone production, (ii) the relative impact of genomic background, evolutionary history and feeding preference in shaping the volatile pheromone bouquet. With respect to HMP research, it remains to be determined: (i) whether HMP is produced by specific glands, and how it is produced, (ii) which genes and pathways are involved in its synthesis, (iii) which is the chemical composition of most HMPs; although their widespread presence in tephritids, these pheromones were so far characterised in very few species; thus, expanding their characterisation represents a major challenge; (iv) whether HMPs are really absent in the *Bactrocera* genus. Although CHs have been widely described and novel studies are continuously being published, we still do not know which is their exact function in true fruit fly mating. Providing an answer to these and to several other questions that may arise when diving into this multifaceted field will be essential for the implementation of novel/improved approaches to tephritid pest control. Indeed, a series of knowledge gaps do exist, which limit the toolbox in the field. First of all, strategies to improve the formulation of semiochemical-based lures currently used in field applications are needed. These include the optimisation of liquid and solid dispensers containing individual compounds or mixtures (including isomers). Moreover, no male lures are currently available for *Anastrepha* and *Rhagoletis* spp., and some *Bactrocera* and *Ceratitis* species do not respond to the existing substances. Alternative and species-specific attractants are needed and the example of the lactone-baited traps used to capture *R. completa* is stimulating further studies aiming at exploring volatile pheromones for trapping. In addition to the MAT-based approaches, other strategies able to interfere with mating need to be explored, including the potential applications of CHs as disruptors. Overall, expanding our understanding of the identity of HMPs will facilitate the integration of multiple approaches for fruit fly control, including biological control, with special reference to the programs relying on the use of parasitoids. Finally, novel technologies allowing the production of nanosensors able to specifically detect pheromone components may open new routes for tephritid pest control.

## Figures and Tables

**Table 1:** List of tephritid species for which volatile pheromone has been chemically analysed from the headspace.

Genus	Species	Male/Female-Borne	References
*Anastrepha*	*A. fraterculus*	Male	[175,176,177,178,179]
	*A. ludens*	Male	[87,180,181,182,183,184,185,186]
	*A. obliqua*	Male	[187,188,189,190,191,192]
	*A. serpentina*	Male	[193]
	*A. suspensa*	Male	[149,180,185,194,195,196,197,198,199,200,201,202,203]
*Bactrocera*	*B. carambolae*	Male	[72]
	*B. dorsalis s.s.*	Male/Female	[68,204,205,206]
	*B. musae*	Male/Female	[76]
	*B. oleae*	Male/Female	[80,174,207,208]
	*B. tryoni*	Male/Female	[209,210,211,212]
	*B. zonata*	Male/Female	[213]
*Zeugodacus*	*Z. cucurbitae*	Male/Female	[204,205]
*Ceratitis*	*C. anonae*	Male	[214]
	*C. capitata*	Male	[166,173,215,216,217,218,219,220,221,222,223,224,225]
	*C. fasciventris*	Male	[214]
	*C. rosa*	Male	[214]
*Rhagoletis*	*R. batava*	Male	[61]
	*R. cerasi*	Male	[226]

**Table 2:** List of tephritid species for which pheromones have been derived from rectal glands extracts.

Genus	Species	Sex	References
*Anastrepha*	*A. fraterculus*	Male *	[227]
	*A. ludens*	Male *	[182]
	*A. ludens*	Male *	[228]
	*A. ludens*	Male *	[183]
	*A. ludens*	Male *	[185]
	*A. ludens*	Male *	[184]
	*A. suspensa*	Male *	[203]
*Bactrocera*	*B. albistrigata*	Male	[229]
	*B. cacuminata*	Male	[230]
	*B. carambolae*	Male	[72]
	*B. correcta*	Male/Female	[231]
	*B. correcta*	Male	[232]
	*B. distincta*	Male	[230]
	*B. dorsalis*	Male	[68]
	*B. dorsalis*	Male	[229]
	*B. facialis*	Male	[233]
	*B. halfordiae*	Male	[234]
	*B. kirki*	Male	[233]
	*B. kraussi*	Male	[233]
	*B. latifrons*	Male	[234]
	*B. musae*	Male/Female	[76]
	*B. neohumeralis*	Male	[209]
	*B. nigrotibialis*	Male	[229]
	*B. occipitalis*	Male	[234]
	*B. oleae*	Male/Female	[79]
	*B. oleae*	Male/Female	[85]
	*B. oleae*	Female	[230]
	*B. oleae*	Male/Female	[85]
	*B. oleae*	Male	[235]
	*B. oleae*	Female	[174]
	*B. passiflorae*	Male	[233]
	*B. tryoni*	Male	[209]
	*B. tryoni*	Female *	[210]
	*B. tryoni*	Female	[211]
	*B. tryoni*	Male	[212]
	*B. tryoni*	Male/Female	[236]
	*B. umbrosa*	Male	[237]
	*B. xanthodes*	Male	[233]
*Zeugodacus*	*Z. cucumis*	Male	[234]
	*Z. cucurbitae*	Male	[238]
	*Z. cucurbitae*	Male	[239]
	*Z. cucurbitae*	Male	[237]
	*Z. tau*	Male	[237]
*Ceratitis*	*-*	-	-
*Rhagoletis*	*-*	-	-

* abdominal extracts.

**Table 3:** List of tephritid species for which host-marking behaviour has been identified.

Genus	Species	Chemical Identity	References
*Anastrepha*	*A. suspensa*	-	[376]
	*A. sororcula*	-	[377]
	*A. fraterculus*	-	[378]
	*A. pseudoparallela*	-	[379]
	*A. bistrigata*	-	[380]
	*A. grandis*	-	[381]
	*A. ludens*	2-(2,14-Dimethylpentadecanoylamino)pentane-dioic acid	[123,382]
	*A. striata*	-	[97]
	*A. obliqua*	-	[132]
	*A. serpentina*	-	[132]
*Bactrocera*	-	-	-
*Ceratitis*	*C. capitata*	-	[111,126]
	*C. cosyra*	Glutathione	[33]
	*C. rosa*	Glutamic acid	[34]
*Rhagoletis*	*R. pomonella*	-	[112,374,383,384]
	*R. cerasi*	*N*-[15(*β*-Glucopyranosyl)-oxy-8-hydroxypalmitoyl]-taurine	[30,385,386]
	*R. completa*	-	[135]
	*R. fausta*	-	[387]
	*R. cingulata*	-	[388]
	*R. cornivora*	-	[388]
	*R. indifferens*	-	[388]
	*R. mendax*	-	[388]
	*R. tabellaria*	-	[388]
	*R. basiola*	-	[130]
	*R. zephyria*	-	[131]
	*R. alternata*	-	[389]
*Zeugodacus*	-	-	-

**Table 4:** List of tephritid species for which CHs have been characterised.

Genus	Species	Developmental Stage	References
*Anastrepha*	*A. ludens*	larvae and adults	[414,415]
	*A. suspensa*	larvae and adults	[414,416,417,418]
	*A. fraterculus*	larvae and adults	[90,93,289,416,417,419]
	*A. acris*	larvae	[416,417]
	*A. obliqua*	larvae and adults	[417]
	*A. serpentina*	larvae	[417]
	*A. pickeli*	larvae	[417]
	*A. striata*	larvae	[417]
*Bactrocera*	*B. dorsalis*	larvae and adults	[414,420,421]
	*B. carambolae*	adults	[421]
	*B. oleae*	adults	[422]
	*B. tryoni*	adults	[423]
	*B. zonata*	adults	[422]
*Zeugodacus*	*Z. cucurbitae*	larvae and adults	[205,414]
*Ceratitis*	*C. capitata*	larvae and adults	[274,289,414,418]
	*C. fasciventris*		[274,289]
	*C. anonae*		[274,289]
	*C. rosa*	larvae and adults	[92,274,289,414]
*Rhagoletis*	-	-	-

## Supplementary Materials

The following are available online at https://www.mdpi.com/article/10.3390/insects12050408/s1, Table S1: Chemicals detected in the volatile pheromone (headspace) of 18 tephritid species of high agricultural relevance. Table S2: List of unique and overlapping chemicals characterised in the volatile pheromone of tephritid species. Table S3: Chemicals with a HMP role detected in the adult female faecal matter of four tephritid species of high agricultural relevance. Table S4: Chemicals detected in the cuticular hexane body washes of 19 tephritid species of high agricultural relevance.

## Abbreviations

The following abbreviations and acronyms are used in this manuscript:

AW-IPM	Area-Wide Integrated Pest Management
CCE	Conventional Chemical Ecology
CHs	Cuticular Hydrocarbons
CI	Chemical Ionization
CL	Cue Lure
CRCE	Computational Reverse Chemical Ecology
CSD	Current Source Density
CSPs	Chemosensory Proteins
DESI	Desorption Electrospray Ionization
DM-HMP	Desmethyl Host-Marking Pheromone
DVB/CAR/PDMS	Divinylbenzene/Carboxen/Polydimethylsiloxane
EAG	Electroantennogram
EI	Electron Ionization
ELSD	Evaporative Light Scattering Detectors
FAR	*Ceratitis* complex including *C. fasciventris*, *C. anonae*, *C. rosa*, and *C. quilici*
GA	Glutamic Acid
GC-EAD	Gas Chromatography-Electroantennographic Detection
GC×GC-TOFMS	Two-dimensional Gas Chromatography with Time-Of-Flight Mass Spectrometry
GC-FID	Gas Chromatography-Flame Ionization Detection
GC-FTIR	Gas Chromatography-Fourier Transformed Infrared Spectroscopy
GC-MS	Gas Chromatography-Mass Spectrometry
GOBP	General OBP
GPCRs	G Protein-Coupled Receptors
GRO	Ginger Root Oil
GRs	Gustatory Receptors
GSH	Tripeptide Glutathione
HMP	Host-Marking Pheromone
HPLC	High Performance Liquid Chromatography
IPM	Integrated Pest Management
IRs	Ionotrophic Receptors
LC-MS	Liquid Chromatography-Mass Spectrometry
LC-QTOF-MS	LC-Quadrupole Time-Of-Flight-Mass Spectrometry
MALDI-TOFMS	Matrix-Assisted Laser Desorption/Ionization-Time-Of-Flight Mass Spectrometry
MAT	Male Annihilation Technique
ME	Methyl Eugenol
MEMS	Microelectromechanical System
MSI	Mass Spectrometric Imaging
NMR	Nuclear Magnetic Resonance
OBPs	Odorant Binding Proteins
Orco	Olfactory receptor co-receptor
ORs	Olfactory Receptors
OSNs	Olfactory Sensory Neurons
PDMS/DVB	Polydimethylsiloxane/Divinylbenzene
RCE	Reverse Chemical Ecology
RK	Raspberry Ketone
RKTA	Raspberry Ketone Trifluoroacetate
RNAi	RNA Interference
SIT	Sterile Insect Technique
SPME	Solid Phase Microextraction
SSR	Single Sensillum Recording
TLC	Thin Layer Chromatography
UV-LDI o-TOFMS	Ultraviolet Laser Desorption Ionization orthogonal Time-Of-Flight Mass Spectrometry
VOCs	Volatile Organic Compounds
1-NPN	*N*-phenylnaphthalen-1-amine
4-DMP	4-allyl-2,6-dimethoxyphenol
